# Achieving the triple endpoint of body weight reduction thresholds, systolic blood pressure reduction ≥5 mmHg and non-HDL cholesterol <130 mg/dL with tirzepatide in people with obesity: A post hoc analysis from the SURMOUNT trials

**DOI:** 10.1371/journal.pone.0345032

**Published:** 2026-08-13

**Authors:** Naveed Sattar, Reshmi Srinath, Luis-Emilio García-Pérez, Clare J. Lee, Chrisanthi A. Karanikas, Xinyue Chen, Arian Plat

**Affiliations:** 1 School of Cardiovascular and Metabolic Health, University of Glasgow, Glasgow, United Kingdom; 2 Icahn School of Medicine at Mount Sinai, New York, New York, United States of America; 3 Eli Lilly and Company, Indianapolis, Indiana, United States of America; All India Institute of Medical Sciences, INDIA

## Abstract

**Background:**

Modest body weight reduction can greatly improve many health-related outcomes. In the SURMOUNT clinical trial program, once-weekly tirzepatide resulted in substantial body weight reductions in people with obesity, without and with type 2 diabetes.

**Objective:**

This post-hoc analysis assessed the proportion of participants achieving body weight reduction thresholds (≥5%, ≥10%, ≥15%), systolic blood pressure reduction ≥5 mmHg, and non-high-density lipoprotein cholesterol (non-HDL-C) <130 mg/dL with tirzepatide.

**Methods:**

Participants from SURMOUNT 1–4 randomized to tirzepatide or placebo with valid baseline and ≥1 non-missing post-baseline body weight, systolic blood pressure and non-HDL-C measurements were included in this analysis. The proportion of participants achieving the triple endpoints was assessed at the primary endpoint of each study (Week 72 for SURMOUNT-1, SURMOUNT-2 and SURMOUNT-3 and Week 88 for SURMOUNT-4).

**Results:**

Across the trials, 32–38% vs 2–8% of tirzepatide-treated vs placebo-treated participants achieved ≥5% body weight reduction, systolic blood pressure reduction ≥5 mmHg, and non-HDL-C <130 mg/dL, 28–37% vs 1–5%, respectively, achieved the triple endpoint with the ≥10% body weight reduction threshold, and 22–34% vs 1–3%, respectively, achieved the triple endpoint with the ≥15% body weight reduction threshold. The between-treatment odds ratios for these proportions across the trials, were statistically significant (all p<0.001).

**Conclusion:**

In this post hoc analysis of SURMOUNT, more participants achieved the triple endpoint of body weight reduction thresholds, systolic blood pressure reduction ≥5 mmHg and non-HDL-C <130 mg/dL with tirzepatide vs placebo. Studies evaluating the potential cardiovascular benefits of tirzepatide are ongoing.

## Introduction

Obesity is a complex condition associated with many chronic diseases [1–4]. Obesity has also been associated with a reduction in disease-free life (ranging from 3–8 years) [5] and with an estimated 1.3-fold higher risk of early death compared to individuals with healthy body weight [6–8].

Modest body weight reductions of 5% may lessen or resolve certain obesity-related complications [9]. In the Look AHEAD study, body weight reductions of 5% to <10% significantly improved glycemic control, lipid levels, and blood pressure in participants with obesity and without type 2 diabetes, and even greater odds of improving cardiovascular risk factors were observed in individuals who achieved 10–15% body weight reduction [10]. As the prevalence of obesity continues to rise, targeted pharmacotherapy in addition to lifestyle modification has become more pertinent to the management of chronic, excess body weight.

Tirzepatide is a once weekly glucose-dependent insulinotropic polypeptide (GIP) and glucagon-like peptide-1 (GLP-1) receptor agonist approved for type 2 diabetes and obesity, among other indications. In the SURMOUNT clinical trial program, tirzepatide treatment at all doses studied (5 mg, 10 mg, or 15 mg or the maximum tolerated dose [MTD] of 10 mg or 15 mg) resulted in robust and clinically meaningful reductions in body weight ranging from 13–26%, with 82–99% of participants achieving body weight reduction thresholds of ≥5% over a period of 72 or 88 weeks in the respective studies [11–14]. Furthermore, significant reductions in systolic blood pressure of up to 11 mmHg and non-high-density lipoprotein cholesterol (non-HDL-C) of up to 13% were observed with tirzepatide compared with placebo [11–14], suggesting improved cardiometabolic health beyond the observed body weight reduction.

Many individuals do not meet the recommended thresholds for these biomarkers [15], which may increase cardiovascular risk. Hence, a broader evaluation of the cardiometabolic changes associated with tirzepatide may offer further evidence of its potential clinically relevant benefits for people living with obesity, with and without type 2 diabetes. Although prespecified cardiovascular outcomes trials for treatment with this dual agonist in people with obesity without type 2 diabetes are ongoing, GLP-1 receptor agonists have been shown to have cardioprotective effects and reduce the risk of major adverse cardiovascular events that may lead to death [16].

Combining multiple endpoints into a single composite can offer a broader perspective on the potential of additional benefits of obesity treatment on related cardiovascular comorbidities. In this post hoc analysis of SURMOUNT, we assessed the proportion of participants who achieved the triple composite endpoint of body weight reduction thresholds (≥5%, ≥10%, ≥15%), with reductions in systolic blood pressure ≥5 mmHg [17] and non-HDL-C <130 mg/dL [18] following treatment with tirzepatide compared with placebo.

## Materials and methods

### Participants and study design

The study designs and primary results of the SURMOUNT-1 through SURMOUNT-4 trials are published [11–14]. Briefly, the SURMOUNT clinical program consisted of Phase 3 randomized controlled trials, ranging from 72 to 88 weeks in duration and designed to assess tirzepatide 5 mg, 10 mg, and 15 mg or MTD of 10 mg and 15 mg in adults, obesity (body mass index [BMI] ≥30 kg/m^2^) or overweight (BMI ≥27 kg/m^2^ with at least one weight-related comorbid condition), without and with type 2 diabetes [11–14]. SURMOUNT-4 included a 36-week, open-label lead-in period with tirzepatide treatment followed by a 52-week, double-blind, placebo-controlled period [14]. In all trials, the primary endpoint was the percentage change in body weight from randomization to end of treatment, with superiority of tirzepatide vs placebo. Due to the design differences of these SURMOUNT trials, they were analyzed separately in this study. Given that participants in the placebo arm of SURMOUNT-4 received tirzepatide in the 36-week lead-in period prior to being randomized, this placebo arm was expected to outperform the placebo arm of the other SURMOUNT trials.

The SURMOUNT clinical trials included in this analysis are registered in clinicaltrials.gov (NCT04184622 [SURMOUNT-1], NCT04657003 [SURMOUNT-2], NCT04657016 [SURMOUNT-3], and NCT04660643 [SURMOUNT-4]) and were conducted in accordance with the International Conference on Harmonization Guidelines for Good Clinical Practice and the Declaration of Helsinki. All participants provided signed informed consent, and protocols were approved by local ethical review boards. Eli Lilly and Company (Indianapolis, IN, USA) sponsored the program.

### Endpoints

The triple composite endpoints consisted of participants achieving a body weight reduction ≥5% with systolic blood pressure reduction ≥5 mmHg and non-HDL-C <130 mg/dL, a body weight reduction ≥10% with systolic blood pressure reduction ≥5 mmHg and non-HDL-C <130 mg/dL, and a body weight reduction ≥15% with systolic blood pressure reduction ≥5 mmHg and non-HDL-C <130 mg/dL. End of treatment body weight, absolute and percent changes from baseline in body weight, systolic blood pressure, and non-HDL-C (overall population and by baseline non-HDL-C <130 mg/dL and ≥130 mg/dL) were evaluated at the primary endpoint of each respective trial (Week 72 for SURMOUNT-1, SURMOUNT-2 and SURMOUNT-3 and Week 88 for SURMOUNT-4).

### Statistical analyses

Only participants with valid baseline body weight and at least one non-missing post-baseline body weight, with valid baseline systolic blood pressure and at least one non-missing post-baseline systolic blood pressure, and with valid baseline non-HDL-C and at least one non-missing post-baseline non-HDL-C were included in this analysis. The proportion of participants achieving the triple composite endpoints was compared between the tirzepatide (SURMOUNT-1: 5 mg, 10 mg or 15 mg, SURMOUNT-2: 10 mg or 15 mg, SURMOUNT-3 and SURMOUNT-4: MTD of 10 mg or 15 mg) and placebo groups (including participants from SURMOUNT-4 who underwent a 36-week lead-in with tirzepatide prior to switching to placebo) using the efficacy analysis dataset. The efficacy analysis dataset included on-treatment data from randomly assigned participants who were exposed to at least one dose of the study drug. Participants who discontinued the study drug because of inadvertent enrolment were excluded from the analysis. Missing endpoint measures for body weight, systolic blood pressure, and non-HDL-C were imputed separately using a mixed-model repeated measures analysis. The imputed values were used to determine whether each component threshold was met, and the resulting composite endpoint status was analyzed using the logistic regression adjusted for relevant baseline values and stratification factors. In SURMOUNT-1, a logistic regression model was fitted with treatment group (placebo, tirzepatide 5 mg, 10 mg, 15 mg) as a factor, consistent with the fixed-dose parallel-group design. Similarly, in SURMOUNT-2, a logistic regression model was fitted with treatment group (placebo, tirzepatide 10 mg, and 15 mg) as a factor. In SURMOUNT-3 and SURMOUNT-4, tirzepatide was administered as a maximum tolerated dose (MTD) and therefore a single comparison of MTD versus placebo was conducted. Safety data were descriptively assessed.

All analyses presented herein were exploratory in nature, thus p-values were nominal, and multiplicity adjustment was not performed. The statistical significance threshold of a p-value <0.05 was applied to assist data interpretation and functioned as hypothesis-generating. Analyses were performed using SAS version 9.4 (Copyright © 2017 SAS Institute Inc., Cary, NC, USA). Additional statistical analysis methods are provided in the **S1 Appendix**. The data sets used in this analysis are provided in the **S2 Appendix**.

## Results

### Participants

The numbers of participants included in this analysis were N = 2345, N = 879, N = 515, and N = 570 in SURMOUNT-1 through SURMOUNT-4, respectively. Overall, baseline demographics and clinical characteristics were similar between treatment groups within trials (Table 1). Baseline demographics and clinical characteristics in participants by achievement of each composite endpoint (yes, no) are presented in **S1**, **S2** **and** **S3 Tables**.

**Table 1:** Baseline demographics and clinical characteristics.

Parameter	SURMOUNT-1 (N = 2345)	SURMOUNT-2 (N = 879)	SURMOUNT-3 (N = 515)	SURMOUNT-4 (N = 570)
**Age, years**	45.0 ± 12.4	54.2 ± 10.4	45.8 ± 12.2	48.0 ± 12.7
**Sex**				
Male	759 (32)	431 (49)	189 (37)	170 (30)
Female	1586 (68)	448 (51)	326 (63)	400 (70)
**Race**				
American Indian or Alaska Native	226 (10)	0	6 (1)	0
Asian	260 (11)	119 (14)	2 (0.4)	45 (8)
Black or African American	177 (8)	71 (8)	53 (10)	64 (11)
Native Hawaiian or other Pacific Islander	8 (0.3)	2 (0.2)	0	2 (0.4)
White	1644 (70)	665 (76)	446 (87)	453 (80)
Multiple	30 (1)	22 (3)	8 (2)	6 (1)
**Ethnicity**				
Hispanic or Latino	1133 (48)	525 (60)	283 (55)	253 (44)
Not Hispanic or Latino	1022 (44)	336 (38)	227 (44)	316 (55)
Not reported	190 (8)	18 (2)	5 (1)	1 (0.2)
**Duration of obesity, years**	14.4 ± 10.8	17.7 ± 11.6	15.3 ± 11.2	15.8 ± 12.0
**Body weight, kg**	104.7 ± 22.1	100.8 ± 21.1	101.5 ± 21.1	106.8 ± 21.8
**Body mass index, kg/m** ^**2**^	38.0 ± 6.8	36.1 ± 6.6	35.9 ± 6.3	38.3 ± 6.4
**Waist circumference, cm**	113.9 ± 15.1	115.0 ± 14.3	109.3 ± 15.0	115.0 ± 14.5
**Duration of type 2 diabetes, years**	N/A	8.5 ± 6.5	N/A	N/A
**Glycated hemoglobin, %**	N/A	8.02 ± 0.89	5.35 ± 0.39	5.54 ± 0.35
**Fasting serum glucose, mg/dL**	N/A	159.2 ± 45.9	91.9 ± 10.6	N/A
**Systolic blood pressure, mmHg**	123.4 ± 12.7	130.6 ± 12.2	120.8 ± 12.7	126.3 ± 13.1
**Diastolic blood pressure, mmHg**	79.6 ± 8.1	79.8 ± 8.5	78.3 ± 9.1	80.9 ± 8.2
**Pulse rate, bpm**	72.3 ± 9.6	75.5 ± 10.0	70.9 ± 10.6	72.0 ± 9.3
**eGFR (CKD-EPI), mL/min/1.73 m** ^**2**^	97.9 ± 17.8	95.1 ± 18.1	96.2 ± 16.8	97.5 ± 17.2
**Total cholesterol, mg/dL**	187 (86, 410)	175 (80, 350)	182 (87, 336)	190 (84, 454)
**Non-HDL-C, mg/dL**	139 (50, 384)	129 (27, 331)	132 (52, 286)	139 (24, 366)
**HDL-C, mg/dL**	47 (16, 126)	43 (15, 91)	47 (23, 118)	50 (25, 100)
**LDL-C, mg/dL**	111 (16, 308)	95 (4, 242)	108 (25, 262)	112 (3, 292)
**VLDL-C, mg/dL**	58 (14, 183)	71 (19, 183)	50 (19, 163)	52 (19, 183)
**Triglycerides, mg/dL**	126 (31, 2845)	157 (42, 1896)	110 (39, 480)	117 (38, 614)
**Prediabetes**	949 (41)	N/A	N/A	N/A
**Hypertension**	756 (32)	N/A	181 (35)	206 (36)
**Dyslipidemia**	698 (30)	N/A	136 (26)	185 (33)
**Obstructive sleep apnea**	180 (8)	N/A	53 (10)	67 (12)
**Atherosclerotic cardiovascular disease**	73 (3)	N/A	10 (2)	34 (6)

Data are mean ± standard deviation or n (%). Lipids are median (min, max). Note: Data are from all randomized participants who took at least one dose of study drug (modified intention-to-treat population) from the efficacy analysis set.

Abbreviations: CKD-EPI = chronic kidney disease epidemiology collaboration; eGFR = estimated glomerular filtration rate; HDL-C = high-density lipoprotein cholesterol; LDL-C = low-density lipoprotein cholesterol; N/A = not available or applicable; VLDL-C = very low-density lipoprotein cholesterol.

### Triple composite endpoints

**Fig 1:**
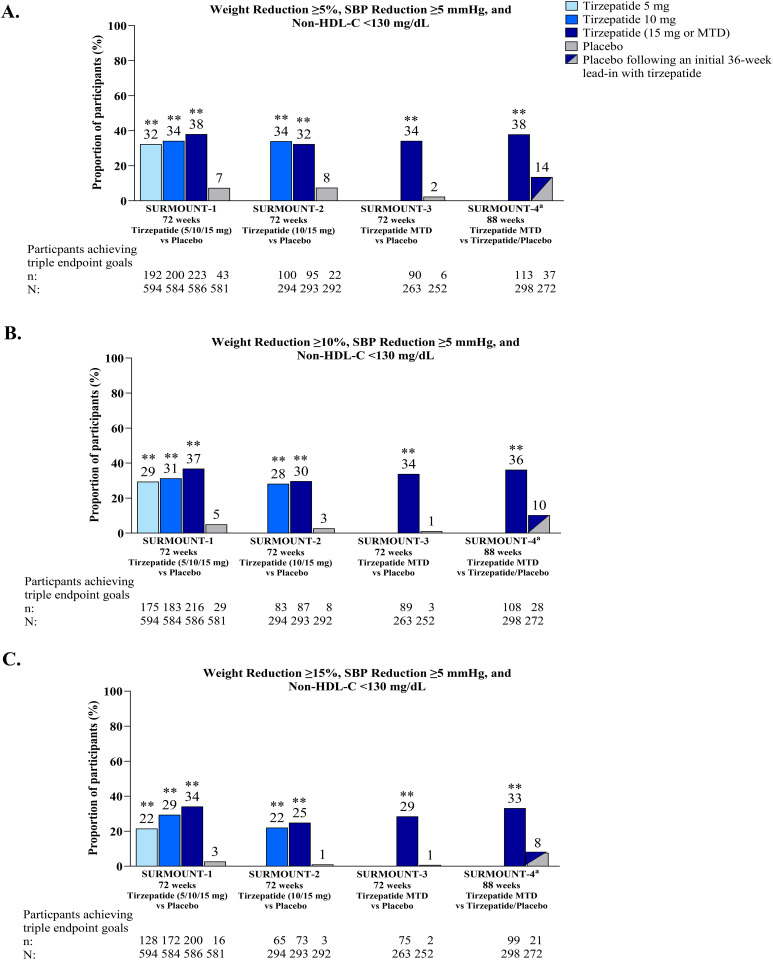
Proportions of participants achieving the triple composite endpoints Data are proportion of participants achieving triple composite endpoints at Week (SURMOUNT 1-3) and Week 88 (SURMOUNT-4) from the modified intent-to-treat population (efficacy analysis set). Data included observed values. Missing endpoint measures were imputed by predictions using observed data in the efficacy analysis set from the same treatment group through an MMRM analysis model for post-baseline measures. (A) Proportions of participants achieving the triple composite endpoints of body weight reduction threshold ≥5% with systolic blood pressure reduction ≥5 mmHg and non-HDL-C <130 mg/dL. (B) body weight reduction threshold ≥10% with systolic blood pressure reduction ≥5 mmHg and non-HDL-C <130 mg/dL. (C) body weight reduction threshold ≥15% with systolic blood pressure reduction ≥5 mmHg and non-HDL-C <130 mg/dL. **p<0.001 for tirzepatide versus placebo within each study, based on logistic regression. All pairwise comparisons across all trials and body weight reduction thresholds were statistically significant (all p<0.001). See S5 Table for trial-specific odds ratios, 95% confidence intervals, and p-values. ^a^SURMOUNT-4 study design included a 36-week, open-label tirzepatide lead-in period followed by a 52-week, double-blind, placebo-controlled period [14].

Baseline, change from baseline and percent change from baseline for individual parameters are presented in **S4 Table**. Across SURMOUNT-1 through SURMOUNT-4, a significantly greater proportion of participants treated with tirzepatide at all doses studied achieved all composite endpoints compared with placebo at the primary endpoint of Week 72/88 (all p<0.001). The proportion of participants who achieved body weight reduction ≥5% with systolic blood pressure reduction ≥5 mmHg and non-HDL-C <130 mg/dL with tirzepatide ranged from 32–38% vs 2–8% with placebo (Fig 1A, **S5 Table**). The odds ratios (95% confidence intervals) were 6.87 (4.75, 9.93), 8.05 (5.56, 11.65), and 9.43 (6.53, 13.62), for tirzepatide 5 mg, 10 mg, and 15 mg vs placebo, respectively, in SURMOUNT-1, 6.52 (3.94, 10.79) and 5.79 (3.50, 9.59) for tirzepatide 10 mg and 15 mg vs placebo, respectively, in SURMOUNT-2, and 24.35 (10.53, 56.29) for tirzepatide MTD vs placebo in SURMOUNT-3 (all p<0.001). The proportion of participants who achieved body weight reduction ≥10% ranged from 28–37% with tirzepatide vs 1–5% with placebo (Fig 1B, **S5 Table**). The odds ratios (95% confidence intervals) were 8.93 (5.86, 13.61), 10.46 (6.86, 15.97), and 13.45 (8.84, 20.47) for tirzepatide 5 mg, 10 mg, and 15 mg vs placebo, respectively, in SURMOUNT-1, 13.47 (6.54, 27.76) and 14.42 (7.00, 29.71) for tirzepatide 10 mg and 15 mg vs placebo, respectively, in SURMOUNT-2, and 45.07 (15.15, 134.06) for tirzepatide MTD vs placebo in SURMOUNT-3 (all p<0.001). The proportion of participants who achieved body weight reduction ≥15% ranged from 22–34% with tirzepatide vs 1–3% with placebo (Fig 1C, **S5 Table**). Odds ratios (95% confidence intervals) were 10.15 (5.97, 17.26), 16.87 (9.96, 28.58), and 20.70 (12.25, 34.97) for tirzepatide 5 mg, 10 mg, and 15 mg vs placebo, respectively, in SURMOUNT-1, 23.57 (8.11, 68.48) and 27.50 (9.48, 79.77) for tirzepatide 10 mg and 15 mg vs placebo, respectively, in SURMOUNT-2, and 45.64 (12.94, 160.92) for tirzepatide MTD vs placebo in SURMOUNT-3 (all p<0.001). In SURMOUNT-4, in participants who switched to placebo following a 36-week lead-in with tirzepatide, 14%, 10% and 8% achieved the composite endpoints with a body weight reduction ≥5%, ≥10%, ≥15%, respectively (Fig 1A-1C, **S5 Table**). The odds ratios (95% confidence intervals) for achieving each composite endpoint in SURMOUNT-4 were 4.58 (2.91, 7.20), 6.05 (3.69, 9.94), and 7.29 (4.24, 12.53), respectively, for tirzepatide MTD vs placebo (all p<0.001).

### Safety

Across the population analyzed, at least one treatment-emergent adverse event was reported by 58–82% of participants treated with tirzepatide or placebo (Table 2). Other than coronavirus disease of 2019, the most frequently reported treatment-emergent adverse events were gastrointestinal-related, including nausea, diarrhea, and constipation.

**Table 2:** Safety profile.

	SURMOUNT-1 N = 2345	SURMOUNT-2 N = 879	SURMOUNT-3 N = 515	SURMOUNT-4 N = 570
**Treatment-emergent adverse events occurring in ≥1% of participants**	1848 (79)	661 (75)	424 (82)	333 (58)
**Treatment-emergent adverse events occurring in ≥5% of participants**				
Nausea	574 (25)	136 (16)	141 (27)	30 (5)
Diarrhea	411 (18)	146 (17)	107 (21)	40 (7)
coronavirus disease of 2019	343 (15)	135 (15)	126 (25)	85 (15)
Constipation	303 (13)	62 (7)	78 (15)	---
Dyspepsia	197 (8)	53 (6)	32 (6)	---
Decreased appetite	192 (8)	65 (7)	35 (7)	---
Vomiting	191 (8)	80 (9)	51 (10)	---
Hyperglycemia	---	52 (6)	---	---
Influenza	---	---	37 (7)	22 (4)
Upper respiratory tract infection	---	42 (5)	41 (8)	22 (4)
Abdominal pain	---	36 (4)	34 (7)	---
Headache	156 (7)	35 (4)	43 (8)	---
Urinary tract infection	106 (5)	---	24 (5)	---
Alopecia	101 (4)	---	23 (5)	---
Back pain	---	34 (4)	27 (5)	---
Eructation	89 (4)	34 (4)	18 (4)	---
Fatigue	---	---	25 (5)	---
Flatulence	---	---	25 (5)	---
Gastroesophageal reflux disease	---	---	25 (5)	---
Nasopharyngitis	---	34 (4)	25 (5)	---
Dizziness	88 (4)	27 (3)	23 (5)	---
Injection site reaction	82 (4)	---	31 (6)	---
Anxiety	---	---	24 (5)	---
Arthralgia	---	---	21 (4)	---
Sinusitis	---	---	21 (4)	---
Ligament sprain	---	---	16 (3)	---

Data are n (%) in participants treated with tirzepatide (5 mg, 10 mg, or 15 mg in SURMOUNT-1, 10 mg or 15 mg in SURMOUNT-2, or MTD in SURMOUNT-3 and SURMOUNT-4) or placebo (safety analysis set, unless otherwise noted).

Study durations: 72 weeks (SURMOUNT-1, SURMOUNT-2 and SURMOUNT-3) and 88 weeks (SURMOUNT-4).

Abbreviation: MTD = maximum tolerated dose.

## Discussion

In this post hoc analysis of the SURMOUNT trials, triple composite endpoints—weight loss, lower systolic blood pressure, and reduced non-HDL-C—were assessed in participants with obesity or overweight, without and with type 2 diabetes. At weeks 72/88, 22–37% of participants treated with tirzepatide achieved body weight reduction thresholds ≥10% or ≥15%, systolic blood pressure reduction  ≥5 mmHg and non-HDL-C <130 mg/dL compared with placebo.

Current guidelines suggest modest weight loss to reduce or prevent some obesity-related complications, while a reduction of body weight of 10% or more may resolve additional comorbidities [9,19]. This analysis higher body weight reduction thresholds to offer additional insight on participants who achieved these more intensive composite endpoints with tirzepatide. Because participants in the placebo group from SURMOUNT-4 received tirzepatide during the 36-week lead-in period before randomization, it was anticipated that this placebo group would perform better than the placebo groups in other SURMOUNT trials [14]. In the SURMOUNT trials, 41–87% of tirzepatide-treated participants achieved at least a 15% reduction in body weight, compared to 2–24% with placebo [11–14].

Obesity causally contributes to other cardiovascular risk factors, including dyslipidemia, type 2 diabetes, hypertension, and sleep disorders [20] and is strongly related to heart failure and kidney disease, regardless of diabetes status [21]. Thus, intentional reductions in body weight may offer the additional benefit of lowering multimorbidity progression and all-cause mortality [22,23]. Recently, the SURPASS-CVOT confirmed our observations in participants with T2D and atherosclerotic cardiovascular disease, which showed that tirzepatide was noninferior to dulaglutide with respect to a composite of death from cardiovascular causes, myocardial infarction, or stroke [24,25], providing further evidence of the potential cardioprotective effects of tirzepatide.

Herein, we report that up to 38% of participants with obesity or overweight, without and with type 2 diabetes, achieved the triple composite endpoint of body weight reduction thresholds (≥5%, ≥10%, ≥15%), systolic blood pressure reduction ≥5 mmHg, and non-HDL-C <130 mg/dL with tirzepatide. In the population reported herein, cardiometabolic parameters were relatively within normal range at baseline and comparable between those who did vs did not achieve each composite endpoint. This may, in part, influence the achievability of each composite endpoint. Therefore, understanding the baseline parameters and their impact on the individual components of the composite endpoint is key for accurate interpretation and analysis of the composite endpoint.

The association between excess adiposity and elevated blood pressure is widely recognized, with estimates indicating that obesity is responsible for approximately 65–78% of hypertension cases [26,27]. Results from a recent meta-analysis suggest that a reduction of 5 mmHg in systolic blood pressure may lower the risk of major cardiovascular events by ~10%, even in individuals who are within a normal range [17]. Similarly, abnormalities in lipid metabolism were observed in up to 70% of the adults living with obesity [28].

Non-HDL-C appears to be a more accurate indicator of cardiovascular death risk than low-density lipoprotein cholesterol (LDL-C) [29,30]. The optimal non-HDL-C level is 30 mg/dL higher than the desired LDL-C level [31]. For individuals with moderate-to-high risk for atherosclerotic cardiovascular disease (ASCVD), this translates to a non-HDL-C <130 mg/dL [31]. Among individuals with low 10-year ASCVD risk, non-HDL-C ≥160 mg/dL was significantly associated with cardiovascular disease and death due to coronary heart disease [32]. In this group, non-HDL-C levels between 130 and 160 mg/dL were linked to higher cardiovascular disease and coronary heart disease mortality, regardless of other risk factors [32]. Results of the current composite endpoint suggest a clinically meaningful response to therapy beyond the observed body weight reduction with tirzepatide.

Overall, the safety profile of the participants included in this analysis was similar to what was observed in the primary SURMOUNT studies [11–14]. Aside from coronavirus disease of 2019, the most frequently reported adverse events were gastrointestinal-related (nausea, diarrhea, constipation).

A limitation of this analysis was its post hoc nature, therefore not adjusted for multiplicity and is intended to be interpreted solely as exploratory and hypothesis-generating. Furthermore, as a composite endpoint assessing risk factors and absolute values together, caution is recommended in interpreting these results. Additional limitations of this analysis are the different trial designs and durations, including the 36-week open-labeled tirzepatide lead-in in SURMOUNT-4, and trial populations, including individuals without and with prediabetes or type 2 diabetes. However, results from the 72-week studies were generally consistent those observed at Week 88 in SURMOUNT-4. Systolic blood pressure and non-HDL-C in the study population were already relatively normal at baseline, which may have attenuated the observed improvement in these parameters and potentially limiting the generalizability of the results to a higher-risk population.

In conclusion, in this post hoc analysis of the SURMOUNT trials, a greater proportion of participants with obesity or overweight, without and with type 2 diabetes, treated with tirzepatide achieved clinically meaningful composite endpoints, including body thresholds ≥5%, ≥10%, and ≥15% with reductions in systolic blood pressure ≥5 mmHg and non-HDL-C <130 mg/dL than those treated with placebo. These findings may suggest an improvement in cardiometabolic risk factors along with clinically meaningful weight reductions associated with tirzepatide treatment. As current guidelines from major international societies evolve, there is greater focus on taking a complication-centric approach to weight management, where those with more severe/advanced comorbidities may warrant more aggressive care. Early intervention with obesity medications like tirzepatide, which can provide meaningful weight loss and potential improvement in cardiometabolic parameters may be considered for individuals living with obesity to mitigate cardiovascular risk. However, whether the improvements in cardiometabolic risk factors observed with tirzepatide treatment in the current analysis can impact morbidity and mortality in adults with obesity who are at risk of cardiovascular disease is currently being investigated in participants with obesity in the SURMOUNT-MMO trial (NCT05556512).

## Supporting information

S1 Appendix Additional statistical analysis methods. (DOCX)

S2 Appendix Data used to construct figures. (CSV)

S1 Table Baseline demographics and clinical characteristics in participants who achieved the triple composite endpoint with body weight reduction ≥5% (Yes, No). (DOCX)

S2 Table Baseline demographics and clinical characteristics in participants who achieved the triple composite endpoint with body weight reduction ≥10% (Yes, No). (DOCX)

S3 Table Baseline demographics and clinical characteristics in participants who achieved the triple composite endpoint achieved with body weight reduction ≥15% (Yes, No). (DOCX)

S4 Table Change from baseline in body weight, systolic blood pressure, and non-HDL-C at the primary endpoint in the SURMOUNT program. (DOCX)

S5 Table Proportion of participants achieving the triple composite endpoint across the SURMOUNT program. (DOCX)
