# Supplementary material for: Achieving the triple endpoint of body weight reduction thresholds, systolic blood pressure reduction ≥5 mmHg and non-HDL cholesterol <130 mg/dL with tirzepatide in people with obesity: A post hoc analysis from the SURMOUNT trials
Source: PLoS One. 2026 Aug 13;21(8):e0345032. doi: 10.1371/journal.pone.0345032 (PMC13472364; doi:10.1371/journal.pone.0345032)
Supplement: Supplementary file 1 [file pone.0345032.s001.docx]

**S1 APPENDIX**

**ADDITIONAL STATISTICAL ANALYSIS METHODS**

The MMRM imputation operates under a missing-at-random (MAR) assumption. Missing continuous values for body weight, SBP, and non-HDL-C were predicted separately using the MMRM model. An unstructured variance-covariance matrix was specified to model within-participant errors. If the model failed to converge, the following structures were tested in order until convergence was achieved: heterogeneous Toeplitz, heterogeneous first-order autoregressive, heterogeneous compound symmetry, Toeplitz, first-order autoregressive, and compound symmetry. The predicted values were then used to determine whether each component threshold was met, and the resulting binary composite endpoint status was analyzed using logistic regression.

The MMRM and logistic regression model covariates varied by trial, consistent with each trial's original statistical analysis plan:

SURMOUNT-1: baseline body weight, baseline SBP, baseline non-HDL-C, country, sex, prediabetes status at randomization, and treatment.

SURMOUNT-2: baseline body weight, baseline SBP, baseline non-HDL-C, country, sex, type of antihyperglycemic medication (AHM) used at randomization, and treatment.

SURMOUNT-3: baseline body weight, baseline SBP, baseline non-HDL-C, country, sex, and treatment.

SURMOUNT-4: lead-in baseline body weight and treatment.

**S1 TABLE. Baseline demographics and clinical characteristics in participants** **who achieved the triple composite endpoint with body weight reduction ≥5% (Yes, No)**

| **Parameter** | **SURMOUNT-1** | | **SURMOUNT-2** | | **SURMOUNT-3** | | **SURMOUNT-4** | |
| --- | --- | --- | --- | --- | --- | --- | --- | --- |
|  | **Yes (N=658)** | **No (N=1687)** | **Yes (N=217)** | **No (N=662)** | **Yes (N=96)** | **No (N=419)** | **Yes (N=150)** | **No (N=420)** |
| **Age, years** | 44.2 ± 12.8 | 45.4 ± 12.2 | 54.2 ± 10.9 | 54.2 ± 10.2 | 43.9 ± 13.0 | 46.2 ± 11.9 | 49.0 ± 12.9 | 47.7 ± 12.6 |
| **Sex** |  |  |  |  |  |  |  |  |
| Male | 205 (31) | 554 (33) | 102 (47) | 329 (50) | 40 (42) | 149 (36) | 46 (31) | 124 (30) |
| Female | 453 (69) | 1133 (67) | 115 (53) | 333 (50) | 56 (58) | 270 (64) | 104 (69) | 296 (71) |
| **Race** |  |  |  |  |  |  |  |  |
| American Indian or Alaska Native | 55 (8.4) | 171 (10) | 0 | 0 | 1 (1) | 5 (1) | 0 | 0 |
| Asian | 83 (13) | 177 (11) | 35 (16) | 84 (13) | 0 | 2 (1) | 13 (9) | 32 (8) |
| Black or African American | 34 (5) | 143 (9) | 14 (7) | 57 (9) | 12 (13) | 41 (10) | 9 (6) | 55 (13) |
| Native Hawaiian or other Pacific Islander | 3 (1) | 5 (0.3) | 0 | 2 (0.3) | 0 | 0 | 1 (1) | 1 (0.2) |
| White | 474 (72) | 1170 (69) | 161 (74) | 504 (76) | 79 (82) | 367 (88) | 124 (83) | 329 (78) |
| Multiple | 9 (1) | 21 (1) | 7 (3) | 15 (2) | 4 (4) | 4 (1) | 3 (2) | 3 (1) |
| **Ethnicity** |  |  |  |  |  |  |  |  |
| Hispanic or Latino | 300 (46) | 833 (49) | 130 (60) | 395 (60) | 54 (56) | 229 (55) | 70 (47) | 183 (44) |
| Not Hispanic or Latino | 288 (44) | 734 (44) | 81 (37) | 255 (39) | 41 (43) | 186 (44) | 80 (53) | 236 (56) |
| Not reported | 70 (11) | 120 (7) | 6 (3) | 12 (2) | 1 (1) | 4 (1) | 0 | 1 (0.2) |
| **Duration of obesity, years** | 14.2 ± 11.3 | 14.4 ± 10.6 | 18.1 ± 11.9 | 17.6 ± 11.5 | 16.5 ± 11.5 | 15.1 ± 11.1 | 15.3 ± 11.2 | 15.9 ± 12.3 |
| **Body weight, kg** | 105.0 ± 21.9 | 104.6 ± 22.1 | 99.6 ± 19.1 | 101.2 ± 21.7 | 102.2 ± 20.9 | 101.3 ± 21.2 | 106.1 ± 21.4 | 107.1 ± 22.0 |
| **Body mass index, kg/m^2^** | 38.1 ± 6.9 | 37.9 ± 6.8 | 36.1 ± 6.3 | 36.1 ± 6.7 | 35.7 ± 5.7 | 35.9 ± 6.4 | 37.9 ± 6.4 | 38.4 ± 6.4 |
| **Waist circumference, cm** | 113.7 ± 14.8 | 114.0 ± 15.3 | 114.8 ± 13.0 | 115.1 ± 14.7 | 108.7 ± 15.2 | 109.4 ± 15.0 | 115.7 ± 14.0 | 114.8 ± 14.8 |
| **Duration of type 2 diabetes, years** | N/A | N/A | 8.4 ± 7.6 | 8.6 ± 6.1 | N/A | N/A | N/A | N/A |
| **Glycated hemoglobin, %** | N/A | N/A | 7.90 ± 0.83 | 8.06 ± 0.90 | 5.30 ± 0.41 | 5.36 ± 0.39 | 5.54 ± 0.32 | 5.54 ± 0.36 |
| **Fasting serum glucose, mg/dL** | N/A | N/A | 155.6 ± 41.2 | 160.3 ± 47.3 | 89.8 ± 10.6 | 92.4 ± 10.6 | N/A | N/A |
| **Systolic blood pressure, mmHg** | 126.2 ± 12.4 | 122.3 ± 12.6 | 132.9 ± 11.5 | 129.9 ± 12.3 | 125.2 ± 11.3 | 119.8 ± 12.8 | 130.5 ± 11.6 | 124.8 ± 13.3 |
| **Diastolic blood pressure, mmHg** | 80.9 ± 7.8 | 79.1 ± 8.2 | 80.5 ± 8.2 | 79.5 ± 8.5 | 80.0 ± 9.0 | 77.9 ± 9.1 | 81.9 ± 7.6 | 80.5 ± 8.4 |
| **Pulse rate, bpm** | 71.5 ± 9.6 | 72.5 ± 9.6 | 74.6 ± 10.3 | 75.8 ± 9.9 | 69.0 ± 10.3 | 71.4 ± 10.6 | 73.0 ± 9.4 | 71.7 ± 9.3 |
| **eGFR (CKD-EPI), mL/min/1.73 m^2^** | 99.1 ± 17.8 | 97.4 ± 17.8 | 95.2 ± 17.6 | 95.1 ± 18.2 | 96.3 ± 17.7 | 96.2 ± 16.6 | 97.0 ± 16.4 | 97.7 ± 17.4 |
| **Total cholesterol, mg/dL** | 176 (91, 350) | 193 (86, 410) | 162 (92, 313) | 181 (80, 350) | 172 (113, 322) | 184 (87, 336) | 174 (106, 280) | 195 (84, 454) |
| **Non-HDL-C, mg/dL** | 125 (55, 295) | 146 (50, 384) | 115 (51, 257) | 137 (27, 331) | 121 (59, 241) | 135 (52, 286) | 125 (71, 212) | 144 (24, 366) |
| Non-HDL-C <130 mg/dL, n (%) | 373 (57) | 573 (34) | 153 (71) | 298 (45) | 60 (63) | 192 (46) | 89 (59) | 149 (36) |
| Non-HDL-C ≥130 mg/dL, n (%) | 285 (43) | 1114 (66) | 64 (30) | 364 (55) | 36 (38) | 227 (54) | 61 (41) | 271 (65) |
| **HDL-C, mg/dL** | 47 (16, 126) | 47 (21, 125) | 43 (19, 79) | 42 (15, 91) | 47 (29, 118) | 47 (23, 98) | 49 (29, 86) | 50 (25, 100) |
| **LDL-C, mg/dL** | 102 (30, 256) | 116 (16, 308) | 86 (19, 213) | 99 (4, 242) | 102 (37, 222) | 110 (25, 262) | 100 (44, 179) | 117 (3, 292) |
| **VLDL-C, mg/dL** | 52 (14, 183) | 59 (16, 179 | 64 (20, 183) | 73 (19, 177) | 45 (20, 114) | 50 (19, 163) | 58 (19, 183) | 52 (19, 169) |
| **Triglycerides, mg/dL** | 116 (31, 1950) | 131 (34, 2845) | 140 (45, 480) | 162 (42, 1896) | 97 (43, 248) | 113 (39, 480) | 124 (38, 513) | 115 (40, 614) |
| **Prediabetes** | 259 (39) | 690 (41) | N/A | N/A | N/A | N/A | N/A | N/A |
| **Hypertension** | 192 (29) | 564 (33) | N/A | N/A | 26 (27) | 155 (37) | 60 (40) | 146 (35) |
| **Dyslipidemia** | 180 (27) | 518 (31) | N/A | N/A | 20 (21) | 116 (28) | 46 (31) | 139 (33) |
| **Obstructive sleep apnea** | 49 (7) | 131 (8) | N/A | N/A | 6 (6) | 47 (11) | 20 (13) | 47 (11) |
| **Atherosclerotic cardiovascular disease** | 27 (4) | 46 (3) | N/A | N/A | 1 (1) | 9 (2) | 10 (7) | 24 (6) |

Data are mean ± standard deviation or n (%). Lipids are median (min, max). Note: Data are from all randomized participants who took at least one dose of study drug (modified intention-to-treat population) from the efficacy analysis set.

Abbreviations: CKD-EPI=chronic kidney disease epidemiology collaboration; eGFR=estimated glomerular filtration rate; HDL-C=high-density lipoprotein cholesterol; LDL-C=low-density lipoprotein cholesterol; N/A=not available or applicable; VLDL-C=very low-density lipoprotein cholesterol.

**S2 TABLE. Baseline demographics and clinical characteristics in participants who achieved the triple composite endpoint with body weight reduction ≥10% (Yes, No)**

| **Parameter** | **SURMOUNT-1** | | **SURMOUNT-2** | | **SURMOUNT-3** | | **SURMOUNT-4** | |
| --- | --- | --- | --- | --- | --- | --- | --- | --- |
|  | **Yes (N=603)** | **No (N=1742)** | **Yes (N=178)** | **No (N=701)** | **Yes (N=92)** | **No (N=423)** | **Yes (N=136)** | **No (N=434)** |
| **Age, years** | 44.2 ± 12.7 | 45.3 ± 12.2 | 53.6 ± 11.0 | 54.4 ± 10.2 | 43.3 ± 12.9 | 46.3 ± 11.9 | 49.0 ± 12.9 | 47.7 ± 12.6 |
| **Sex** |  |  |  |  |  |  |  |  |
| Male | 180 (30) | 579 (33) | 79 (44) | 352 (50) | 38 (41) | 151 (36) | 37 (27) | 133 (31) |
| Female | 423 (70) | 1163 (67) | 99 (56) | 349 (50) | 54 (59) | 272 (64) | 99 (73) | 301 (69) |
| **Race** |  |  |  |  |  |  |  |  |
| American Indian or Alaska Native | 45 (8) | 181 (10) | 0 | 0 | 1 (1) | 5 (1.2) | 0 | 0 |
| Asian | 76 (13) | 184 (11) | 29 (16) | 90 (13) | 0 | 2 (1) | 11 (8) | 34 (8) |
| Black or African American | 32 (5) | 145 (8) | 10 (6) | 61 (9) | 11 (12) | 42 (10) | 8 (6) | 56 (13) |
| Native Hawaiian or other Pacific Islander | 3 (1) | 5 (0.3) | 0 | 2 (0.3) | 0 | 0 | 1 (1) | 1 (0.2) |
| White | 439 (73) | 1205 (69) | 133 (75) | 532 (76) | 76 (83) | 370 (88) | 113 (83) | 340 (78) |
| Multiple | 8 (1) | 22 (1) | 6 (3) | 16 (2) | 4 (4) | 4 (1) | 3 (2) | 3 (1) |
| **Ethnicity** |  |  |  |  |  |  |  |  |
| Hispanic or Latino | 274 (45) | 859 (49) | 106 (60) | 419 (60) | 51 (55) | 232 (55) | 65 (48) | 188 (43) |
| Not Hispanic or Latino | 265 (44) | 757 (44) | 67 (38) | 269 (38) | 40 (44) | 187 (44) | 71 (52) | 245 (57) |
| Not reported | 64 (11) | 126 (7) | 5 (3) | 13 (2) | 1 (1) | 4 (1) | 0 | 1 (0.2) |
| **Duration of obesity, years** | 14.3 ± 11.4 | 14.4 ± 10.6 | 18.5 ± 11.7 | 17.5 ± 11.5 | 16.4 ± 11.3 | 15.1 ± 11.1 | 15.5 ± 11.2 | 15.8 ± 12.3 |
| **Body weight, kg** | 104.4 ± 21.5 | 104.8 ± 22.2 | 100.3 ± 19.5 | 100.9 ± 21.5 | 102.3 ± 21.0 | 101.3 ± 21.2 | 105.7 ± 21.8 | 107.2 ± 21.9 |
| **Body mass index, kg/m^2^** | 38.0 ± 6.6 | 38.0 ± 6.9 | 36.4 ± 6.3 | 36.1 ± 6.7 | 35.7 ± 5.7 | 35.9 ± 6.4 | 38.0 ± 6.5 | 38.4 ± 6.4 |
| **Waist circumference, cm** | 113.3 ± 14.4 | 114.2 ± 15.4 | 115.3 ± 13.2 | 114.9 ± 14.6 | 108.9 ± 15.2 | 109.4 ± 15.0 | 115.8 ± 14.2 | 114.8 ± 14.7 |
| **Duration of type 2 diabetes, years** | N/A | N/A | 8.1 ± 6.9 | 8.7 ± 6.4 | N/A | N/A | N/A | N/A |
| **Glycated hemoglobin, %** | N/A | N/A | 7.83 ± 0.80 | 8.07 ± 0.90 | 5.30 ± 0.41 | 5.36 ± 0.39 | 5.55 ± 0.33 | 5.53 ± 0.36 |
| **Fasting serum glucose, mg/dL** | N/A | N/A | 154.7 ± 40.1 | 160.3 ± 47.2 | 89.8 ± 10.7 | 92.3 ± 10.6 | N/A | N/A |
| **Systolic blood pressure, mmHg** | 126.1 ± 12.5 | 122.5 ± 12.6 | 132.8 ± 11.5 | 130.0 ± 12.3 | 125.4 ± 11.4 | 119.8 ± 12.7 | 130.5 ± 11.4 | 125.0 ± 13.3 |
| **Diastolic blood pressure, mmHg** | 80.8 ± 7.8 | 79.2 ± 8.2 | 80.9 ± 8.3 | 79.5 ± 8.5 | 80.5 ± 8.8 | 77.8 ± 9.1 | 81.9 ± 7.5 | 80.5 ± 8.4 |
| **Pulse rate, bpm** | 71.6 ± 9.7 | 72.5 ± 9.5 | 74.8 ± 9.9 | 75.7 ± 10.1 | 69.5 ± 10.2 | 71.2 ± 10.7 | 72.8 ± 9.2 | 71.8 ± 9.3 |
| **eGFR (CKD-EPI), mL/min/1.73 m^2^** | 99.0 ± 17.7 | 97.5 ± 17.8 | 95.4 ± 17.8 | 95.1 ± 18.1 | 96.6 ± 17.9 | 96.1 ± 16.5 | 97.2 ± 16.3 | 97.7 ± 17.5 |
| **Total cholesterol, mg/dL** | 177 (91, 350) | 193 (86, 410) | 164 (92, 313) | 179 (80, 350) | 173 (113, 322) | 184 (87, 336) | 176 (106, 280) | 194 (84, 454) |
| **Non-HDL-C, mg/dL** | 126 (55, 295) | 144 (50, 384) | 118 (51, 257) | 134 (27, 331) | 123 (59, 241) | 134 (52, 286) | 126 (71, 212) | 143 (24, 366) |
| Non-HDL-C <130 mg/dL, n (%) | 338 (56) | 608 (35) | 119 (67) | 332 (47) | 56 (61) | 196 (46) | 77 (57) | 161 (37) |
| Non-HDL-C ≥130 mg/dL, n (%) | 265 (44) | 1134 (65) | 59 (33) | 369 (53) | 36 (39) | 65 (44) | 59 (43) | 273 (63) |
| **HDL-C, mg/dL** | 47 (21, 126) | 47 (16, 125) | 44 (19, 79) | 42 (15, 91) | 47 (29, 118) | 47 (23, 98) | 49 (29, 86) | 50 (25, 100) |
| **LDL-C, mg/dL** | 102 (30, 256) | 116 (16, 308) | 88 (19, 213) | 98 (4, 242) | 102 (37, 222) | 110 (25, 262) | 100 (44, 179) | 116 (3, 292) |
| **VLDL-C, mg/dL** | 52 (14, 183) | 59 (16, 179) | 64 (20, 183) | 73 (19, 177) | 46 (20, 114) | 50 (19, 163) | 58 (19, 183) | 52 (19, 169) |
| **Triglycerides, mg/dL** | 116 (31, 1950) | 131 (34, 2845) | 141 (45, 480) | 161 (42, 1896) | 100 (43, 248) | 112 (39, 480) | 124 (38, 513) | 115 (40, 614) |
| **Prediabetes** | 236 (39) | 713 (41) | N/A | N/A | N/A | N/A | N/A | N/A |
| **Hypertension** | 178 (30) | 578 (33) | N/A | N/A | 24 (26) | 157 (37) | 53 (39.0) | 153 (35) |
| **Dyslipidemia** | 162 (27) | 536 (31) | N/A | N/A | 19 (21) | 117 (28) | 40 (29.4) | 145 (33) |
| **Obstructive sleep apnea** | 41 (7) | 139 (8) | N/A | N/A | 5 (5) | 48 (11) | 18 (13) | 49 (11) |
| **Atherosclerotic cardiovascular disease** | 23 (4) | 50 (3) | N/A | N/A | 1 (1) | 9 (2) | 9 (7) | 25 (6) |

Data are mean ± standard deviation or n (%). Lipids are median (min, max). Note: Data are from all randomized participants who took at least one dose of study drug (modified intention-to-treat population) from the efficacy analysis set.

Abbreviations: CKD-EPI=chronic kidney disease epidemiology collaboration; eGFR=estimated glomerular filtration rate; HDL-C=high-density lipoprotein cholesterol; LDL-C=low-density lipoprotein cholesterol; N/A=not available or applicable; VLDL-C=very low-density lipoprotein cholesterol.

**S3 TABLE. Baseline demographics and clinical characteristics in participants who achieved the triple composite endpoint achieved with body weight reduction ≥15% (Yes, No)**

| **Parameter** | **SURMOUNT-1** | | **SURMOUNT-2** | | **SURMOUNT-3** | | **SURMOUNT-4** | |
| --- | --- | --- | --- | --- | --- | --- | --- | --- |
|  | **Yes (N=516)** | **No (N=1829)** | **Yes (N=141)** | **No (N=738)** | **Yes (N=77)** | **No (N=438)** | **Yes (N=120)** | **No (N=450)** |
| **Age, years** | 43.8 ± 12.5 | 45.4 ± 12.3 | 53.5 ± 11.0 | 54.3 ± 10.3 | 43.1 ± 11.9 | 46.3 ± 12.2 | 48.9 ± 12.8 | 47.8 ± 12.7 |
| **Sex** |  |  |  |  |  |  |  |  |
| Male | 140 (27) | 619 (34) | 60 (43) | 371 (50) | 27 (35) | 162 (37) | 34 (28) | 136 (30) |
| Female | 376 (73) | 1210 (66) | 81 (57) | 367 (50) | 50 (65) | 276 (63) | 86 (72) | 314 (70) |
| **Race** |  |  |  |  |  |  |  |  |
| American Indian or Alaska Native | 40 (8) | 186 (10) | 0 | 0 | 1 (1) | 5 (1) | 0 | 0 |
| Asian | 59 (11) | 201 (11) | 19 (14) | 100 (14) | 0 | 2 (1) | 8 (7) | 37 (8) |
| Black or African American | 29 (6) | 148 (8) | 8 (6) | 63 (9) | 9 (12) | 44 (10) | 6 (5) | 58 (13) |
| Native Hawaiian or other Pacific Islander | 3 (1) | 5 (0.3) | 0 | 2 (0.3) | 0 | 0 | 1 (1) | 1 (0.2) |
| White | 378 (73) | 1266 (69) | 108 (77) | 557 (76) | 64 (83) | 382 (87) | 102 (85) | 351 (78) |
| Multiple | 7 (1) | 23 (1) | 6 (4) | 16 (2) | 3 (4) | 5 (1) | 3 (3) | 3 (1) |
| **Ethnicity** |  |  |  |  |  |  |  |  |
| Hispanic or Latino | 232 (45) | 901 (49) | 86 (61) | 439 (60) | 41 (53) | 242 (55) | 60 (50) | 193 (43) |
| Not Hispanic or Latino | 236 (46) | 786 (43) | 50 (36) | 286 (39) | 35 (46) | 192 (44) | 60 (50) | 256 (57) |
| Not reported | 48 (9) | 142 (8) | 5 (4) | 13 (2) | 1 (1) | 4 (1) | 0 | 1 (0.2) |
| **Duration of obesity, years** | 14.2 ± 11.4 | 14.4 ± 10.6 | 18.9 ± 12.0 | 17.5 ± 11.5 | 16.7 ± 10.8 | 15.1 ± 11.2 | 15.6 ± 10.9 | 15.8 ± 12.3 |
| **Body weight, kg** | 104.2 ± 21.3 | 104.8 ± 22.3 | 100.2 ± 19.0 | 100.9 ± 21.5 | 102.0 ± 22.1 | 101.4 ± 21.0 | 106.0 ± 20.2 | 107.1 ± 22.3 |
| **Body mass index, kg/m^2^** | 38.0 ± 6.4 | 38.0 ± 6.9 | 36.4 ± 6.1 | 36.1 ± 6.7 | 35.8 ± 5.9 | 35.9 ± 6.4 | 38.0 ± 6.2 | 38.4 ± 6.4 |
| **Waist circumference, cm** | 113.2 ± 14.2 | 114.1 ± 15.4 | 115.6 ± 13.0 | 114.9 ± 14.5 | 108.9 ± 16.0 | 109.4 ± 14.8 | 116.2 ± 12.9 | 114.7 ± 14.9 |
| **Duration of type 2 diabetes, years** | N/A | N/A | 7.6 ± 7.0 | 8.7 ± 6.4 | N/A | N/A | N/A | N/A |
| **Glycated hemoglobin, %** | N/A | N/A | 7.76 ± 0.77 | 8.07 ± 0.90 | 5.29 ± 0.42 | 5.36 ± 0.39 | 5.56 ± 0.32 | 5.53 ± 0.36 |
| **Fasting serum glucose, mg/dL** | N/A | N/A | 150.4 ± 40.9 | 160.9 ± 46.6 | 89.8 ± 10.6 | 92.3 ± 10.6 | N/A | N/A |
| **Systolic blood pressure, mmHg** | 125.3 ± 12.4 | 122.9 ± 12.7 | 132.7 ± 11.5 | 130.2 ± 12.2 | 124.9 ± 11.5 | 120.1 ± 12.8 | 129.9 ± 11.3 | 125.3 ± 13.4 |
| **Diastolic blood pressure, mmHg** | 80.5 ± 7.8 | 79.3 ± 8.2 | 81.0 ± 8.2 | 79.5 ± 8.5 | 80.8 ± 8.5 | 77.8 ± 9.2 | 81.8 ±7.7 | 80.6 ± 8.3 |
| **Pulse rate, bpm** | 71.6 ± 9.8 | 72.4 ± 9.5 | 74.8 ± 9.9 | 75.6 ± 10.1 | 69.9 ± 9.8 | 71.1 ± 10.7 | 72.8 ± 8.9 | 71.9 ± 9.4 |
| **eGFR (CKD-EPI), mL/min/1.73 m^2^** | 99.2 ± 17.7 | 97.5 ± 17.8 | 94.8 ± 17.3 | 95.2 ± 18.2 | 95.9 ± 17.3 | 96.3 ± 16.7 | 97.1 ± 16.3 | 97.7 ± 17.4 |
| **Total cholesterol, mg/dL** | 177 (91, 350) | 191 (86, 410) | 164 (92, 313) | 177 (80, 350) | 173 (113, 322) | 184 (87, 336) | 176 (122, 280) | 193 (84, 454) |
| **Non-HDL-C, mg/dL** | 126 (59, 295) | 143 (50, 384) | 118 (51, 257) | 132 (27, 331) | 123 (64, 241) | 134 (52, 286) | 125 (81, 212) | 143 (24, 366) |
| Non-HDL-C <130 mg/dL, n (%) | 288 (56) | 658 (36) | 91 (65) | 360 (49) | 48 (62) | 204 (47) | 68 (57) | 170 (38) |
| Non-HDL-C ≥130 mg/dL, n (%) | 228 (44) | 1171 (64) | 50 (36) | 378 (51) | 29 (38) | 234 (53) | 52 (43) | 280 (62) |
| **HDL-C, mg/dL** | 48 (21, 109) | 47 (16, 126) | 43 (19, 79) | 43 (15, 91) | 47 (29, 118) | 47 (23, 98) | 49 (32, 86) | 50 (25, 100) |
| **LDL-C, mg/dL** | 103 (30, 256) | 115 (16, 308) | 87 (19, 213) | 97 (4, 242) | 102 (51, 222) | 110 (25, 262) | 101 (44, 179) | 115 (3, 292) |
| **VLDL-C, mg/dL** | 52 (14, 183) | 59 (16, 179) | 66 (20, 183) | 73 (19, 177) | 46 (20, 105) | 50 (19, 163) | 56 (19, 183) | 52 (19, 169) |
| **Triglycerides, mg/dL** | 115 (31, 1950) | 130 (34, 2845) | 144 (45, 480) | 160 (42, 1896) | 98 (43, 229) | 111 (39, 480) | 123 (38, 513) | 116 (40, 614) |
| **Prediabetes** | 196 (38) | 753 (41) | N/A | N/A | N/A | N/A | N/A | N/A |
| **Hypertension** | 140 (27) | 616 (34) | N/A | N/A | 20 (26) | 161 (37) | 41 (34) | 165 (37) |
| **Dyslipidemia** | 133 (26) | 565 (31) | N/A | N/A | 18 (23) | 118 (27) | 35 (29) | 150 (33) |
| **Obstructive sleep apnea** | 34 (7) | 146 (8) | N/A | N/A | 3 (4) | 50 (11) | 14 (12) | 53 (12) |
| **Atherosclerotic cardiovascular disease** | 16 (3) | 57 (3) | N/A | N/A | 1 (1) | 9 (2) | 6 (5) | 28 (6) |

Data are mean ± standard deviation or n (%). Lipids are median (min, max). Note: Data are from all randomized participants who took at least one dose of study drug (modified intention-to-treat population) from the efficacy analysis set.

Abbreviations: CKD-EPI=chronic kidney disease epidemiology collaboration; eGFR=estimated glomerular filtration rate; HDL-C=high-density lipoprotein cholesterol; LDL-C=low-density lipoprotein cholesterol; N/A=not available or applicable; VLDL-C=very low-density lipoprotein cholesterol.

**S4 TABLE. Change from baseline in body weight, systolic blood pressure, and non-HDL-C at the primary endpoint in the SURMOUNT program**

| **Parameter** | **SURMOUNT-1 (N=2345)** | | **SURMOUNT-2 (N=879)** | | **SURMOUNT-3 (N=515)** | | **SURMOUNT-4^a^ (N=570)** | |
| --- | --- | --- | --- | --- | --- | --- | --- | --- |
|  | **Tirzepatide** | **Placebo** | **Tirzepatide** | **Placebo** | **Tirzepatide** | **Placebo** | **Tirzepatide** | **Placebo^b^** |
| **Baseline body weight, kg** | 104.7 (22.31) | 104.7 (21.29) | 100.4 (20.44) | 101.5 (22.30) | 102.7 (1.30) | 100.2 (1.33) | 106.5 (1.27) | 107.2 (1.32) |
| Change at Week 72/88 | -20.8 (0.26) | -2.8 (0.46) | -14.6 (0.36) | -3.3 (0.52) | -21.5 (0.65) | 3.0 (0.67) | -27.4 (0.50) | -10.1 (0.52) |
| Percent change at Week 72/88 | -20.1 (0.23) | -2.8 (0.42) | -14.6 (0.35) | -3.4 (0.50) | -21.2 (0.64) | 3.0 (0.66) | -25.7 (0.47) | -9.4 (0.49) |
| **Baseline systolic blood pressure, mmHg** | 123.5 (0.30) | 123.1 (0.53) | 130.4 (12.31) | 131.1 (11.83) | 121.6 (0.78) | 120.6 (0.80) | 126.8 (0.76) | 125.8 (0.79) |
| Change at Week 72/88 | -8.1 (0.27) | -1.4 (0.49) | -7.2 (0.52) | -0.9 (0.75) | -5.7 (0.73) | 4.2 (0.76) | -9.4 (0.89) | -2.3 (0.95) |
| **Baseline non-HDL-C, mg/dL** | 138.2 (0.86) | 137.2 (1.49) | 124.4 (1.65) | 129.8 (2.44) | 131.8 (2.17) | 130.3 (2.19) | 136.3 (2.17) | 134.7 (2.24) |
| Percent change at Week 72/88 | -11.3 (0.47) | -1.8 (0.96) | -6.6 (1.00) | 2.3 (1.57) | -9.8 (1.26) | 5.6 (1.55) | -10.6 (1.46) | -1.8 (1.72) |
| **Baseline non-HDL-C <130 mg/dL** | 108.0 (0.66) | 106.3 (1.15) | 97.9 (1.24) | 99.8 (1.91) | 106.7 (1.68) | 105.3 (1.67) | 107.0 (1.88) | 103.9 (1.92) |
| Percent change at Week 72/88 | -3.9 (0.85) | 6.2 (1.75) | 3.1 (1.53) | 11.6 (2.50) | -1.9 (2.05) | 14.1 (2.45) | -3.7 (3.31) | 5.3 (3.96) |
| **Baseline non-HDL-C ≥130 mg/dL** | 163.7 (0.82) | 161.8 (1.39) | 163.5 (1.61) | 164.3 (2.16) | 160.5 (2.14) | 160.8 (2.22) | 162.2 (1.93) | 162.0 (2.01) |
| Percent change at Week 72/88 | -16.0 (0.56) | -6.8 (1.11) | -16.1 (1.28) | -6.8 (1.94) | -17.0 (1.53) | -2.0 (1.93) | -16.2 (1.73) | -5.5 (2.10) |

Data are mean (standard deviation) at baseline and least squares mean (standard error) change from baseline at the primary endpoint in participants treated with tirzepatide or placebo, unless otherwise noted. Lipid data are estimate (standard error) using log transformation. The primary endpoint was Week 72 in SURMOUNT-1, SURMOUNT-2 and SURMOUNT-3 and Week 88 in SURMOUNT-4.

Note: Tirzepatide groups refer to pooled tirzepatide 5/10/15mg in SURMOUNT-1, pooled tirzepatide 10/15mg in SURMOUNT-2, and maximum tolerated doses of 10 or 15 mg in SURMOUNT-3 and SURMOUNT-4.

^a^ SURMOUNT-4 was a randomized withdrawal study with a 36-week, open-label tirzepatide lead-in period followed by a 52-week, double-blind, placebo-controlled period. Participants who completed the 36-week lead-in period experienced a mean weight reduction of 20.9%.

^b^ Placebo following an initial 36-week lead-in with tirzepatide.

Abbreviations: Non-HDL-C=non-high-density lipoprotein cholesterol.

**S5 TABLE. Proportion of participants achieving the triple composite endpoint across the SURMOUNT program**

| **Study** | **Body weight reduction threshold**  **Treatment** | **Observed n/N (%)** | **Imputed m/M (%)** | **LSM (SE)** | **Odds Ratio (95% CI)** | **p-value** |
| --- | --- | --- | --- | --- | --- | --- |
| **SURMOUNT-1** | **Body weight reduction ≥5%** |  |  |  |  |  |
|  | Tirzepatide 5 mg | 173/522 (33.14) | 192/594 (32.32) | 29.00 (1.97) | 6.87 (4.75, 9.93) | p<0.001 |
|  | Tirzepatide 10 mg | 179/520 (34.42) | 200/584 (34.25) | 32.37 (2.07) | 8.05 (5.56, 11.65) | p<0.001 |
|  | Tirzepatide 15 mg | 201/520 (38.65) | 223/586 (38.05) | 35.95 (2.13) | 9.43 (6.53, 13.62) | p<0.001 |
|  | Placebo | 37/463 (7.99) | 43/581 (7.40) | 5.61 (0.88) | -- | -- |
|  | **Body weight reduction ≥10%** |  |  |  |  |  |
|  | Tirzepatide 5 mg | 159/522 (30.46) | 175/594 (29.46) | 26.24 (1.89) | 8.93 (5.86, 13.61) | p<0.001 |
|  | Tirzepatide 10 mg | 166/520 (31.92) | 183/584 (31.34) | 29.42 (2.00) | 10.46 (6.86, 15.97) | p<0.001 |
|  | Tirzepatide 15 mg | 194/520 (37.31) | 216/586 (36.86) | 34.89 (2.10) | 13.45 (8.84, 20.47) | p<0.001 |
|  | Placebo | 25/463 (5.40) | 29/581 (4.99) | 3.83 (0.72) | -- | -- |
|  | **Body weight reduction ≥15%** |  |  |  |  |  |
|  | Tirzepatide 5 mg | 118/522 (22.61) | 128/594 (21.55) | 18.94 (1.64) | 10.15 (5.97, 17.26) | p<0.001 |
|  | Tirzepatide 10 mg | 156/520 (30.00) | 172/584 (29.45) | 27.98 (1.94) | 16.87 (9.96, 28.58) | p<0.001 |
|  | Tirzepatide 15 mg | 180/520 (34.62) | 200/586 (34.13) | 32.28 (2.03) | 20.70 (12.25, 34.97) | p<0.001 |
|  | Placebo | 13/463 (2.81) | 16/581 (2.75) | 2.25 (0.56) | -- | -- |
| **SURMOUNT-2** | **Body weight reduction ≥5%** |  |  |  |  |  |
|  | Tirzepatide 10 mg | 95/280 (33.93) | 100/294 (34.01) | 32.35 (2.88) | 6.52 (3.94, 10.79) | p<0.001 |
|  | Tirzepatide 15 mg | 85/263 (32.32) | 95/293 (32.42) | 29.82 (2.81) | 5.79 (3.50, 9.59) | p<0.001 |
|  | Placebo | 21/263 (7.98) | 22/292 (7.53) | 6.84 (1.44) | -- | -- |
|  | **Body weight reduction ≥10** |  |  |  |  |  |
|  | Tirzepatide 10 mg | 78/280 (27.86) | 83/294 (28.23) | 27.00 (2.69) | 13.47 (6.54, 27.76) | p<0.001 |
|  | Tirzepatide 15 mg | 78/263 (29.66) | 87/293 (29.69) | 28.36 (2.73) | 14.42 (7.00, 29.71) | p<0.001 |
|  | Placebo | 8/263 (3.04) | 8/292(2.74) | 2.67 (0.90) | -- | -- |
|  | **Body weight reduction ≥15%** |  |  |  |  |  |
|  | Tirzepatide 10 mg | 60/280 (21.43) | 65/294 (22.11) | 21.41 (2.45) | 23.57 (8.11, 68.48) | p<0.001 |
|  | Tirzepatide 15 mg | 67/263 (25.48) | 73/293 (24.91) | 24.12 (2.57) | 27.50 (9.48, 79.77) | p<0.001 |
|  | Placebo | 3/263 (1.14) | 3/292 (1.03) | 1.14 (0.60) | -- | -- |
| **SURMOUNT-3** | **Body weight reduction ≥5%** |  |  |  |  |  |
|  | Tirzepatide MTD (10 or 15 mg) | 75/223 (33.63) | 90/263 (34.22) | 32.43 (3.11) | 24.35 (10.53, 56.29) | p<0.001 |
|  | Placebo | 5/201 (2.49) | 6/252 (2.38) | 1.93 (0.78) | -- | -- |
|  | **Body weight reduction ≥10%** |  |  |  |  |  |
|  | Tirzepatide MTD (10 or 15 mg) | 74/223 (33.18) | 89/263 (33.84) | 32.07 (3.10) | 45.07 (15.15, 134.06) | p<0.001 |
|  | Placebo | 3/201 (1.49) | 3/252 (1.19) | 1.04 (0.56) | -- | -- |
|  | **Body weight reduction ≥15%** |  |  |  |  |  |
|  | Tirzepatide MTD (10 or 15 mg) | 63/223 (28.25) | 75/263 (28.52) | 27.19 (2.90) | 45.64 (12.94, 160.92) | p<0.001 |
|  | Placebo | 2/201 (1.00) | 2/252 (0.79) | 0.81 (0.51) | -- | -- |
| **SURMOUNT-4** | **Body weight reduction ≥5%** |  |  |  |  |  |
|  | Tirzepatide MTD (10 or 15 mg) | 113/298 (37.92) | 113/298 (37.92) | 35.17 (3.02) | 4.58 (2.91, 7.20) | p<0.001 |
|  | Placebo, following an initial 36-week lead-in with tirzepatide | 37/272 (13.60) | 37/272 (13.60) | 10.60 (1.90) | -- | -- |
|  | **Body weight reduction ≥10%** |  |  |  |  |  |
|  | Tirzepatide MTD (10 or 15 mg) | 108/298 (36.24) | 108/298 (36.24) | 33.22 (2.99) | 6.05 (3.69, 9.94) | p<0.001 |
|  | Placebo, following an initial 36-week lead-in with tirzepatide | 28/272 (10.29) | 28/272 (10.29) | 7.59 (1.60) | -- | -- |
|  | **Body weight reduction ≥15%** |  |  |  |  |  |
|  | Tirzepatide MTD (10 or 15 mg) | 99/298 (33.22) | 99/298 (33.22) | 30.21 (2.90) | 7.29 (4.24, 12.53) | p<0.001 |
|  | Placebo, following an initial 36-week lead-in with tirzepatide | 21/272 (7.72) | 21/272 (7.72) | 5.61 (1.36) | -- | -- |

LSM represents model-adjusted attainment rates from logistic regression. Abbreviations: CI=confidence interval; LSM=least-squares mean; M=number of participants in imputed data; m=number of participants achieving target in imputed data; MTD=maximum tolerated dose; N=number of participants with baseline value and at least one non-missing post-baseline value; n=number of participants achieving target in observed data; SE=standard error.
