# Supplementary material for: Achieving the triple endpoint of body weight reduction thresholds, systolic blood pressure reduction ≥5 mmHg and non-HDL cholesterol <130 mg/dL with tirzepatide in people with obesity: A post hoc analysis from the SURMOUNT trials
Source: PLoS One. 2026 Aug 13;21(8):e0345032. doi: 10.1371/journal.pone.0345032 (PMC13472364; doi:10.1371/journal.pone.0345032)
Supplement: Supplementary file 2 [file pone.0345032.s003.docx]

**S1 TABLE. Baseline demographics and clinical characteristics in participants** **who achieved the triple composite endpoint with body weight reduction ≥5% (Yes, No)**

| **Parameter** | **SURMOUNT-1** | | **SURMOUNT-2** | | **SURMOUNT-3** | | **SURMOUNT-4** | |
| --- | --- | --- | --- | --- | --- | --- | --- | --- |
|  | **Yes (N=658)** | **No (N=1687)** | **Yes (N=217)** | **No (N=662)** | **Yes (N=96)** | **No (N=419)** | **Yes (N=150)** | **No (N=420)** |
| **Age, years** | 44.2 ± 12.8 | 45.4 ± 12.2 | 54.2 ± 10.9 | 54.2 ± 10.2 | 43.9 ± 13.0 | 46.2 ± 11.9 | 49.0 ± 12.9 | 47.7 ± 12.6 |
| **Sex** |  |  |  |  |  |  |  |  |
| Male | 205 (31) | 554 (33) | 102 (47) | 329 (50) | 40 (42) | 149 (36) | 46 (31) | 124 (30) |
| Female | 453 (69) | 1133 (67) | 115 (53) | 333 (50) | 56 (58) | 270 (64) | 104 (69) | 296 (71) |
| **Race** |  |  |  |  |  |  |  |  |
| American Indian or Alaska Native | 55 (8.4) | 171 (10) | 0 | 0 | 1 (1) | 5 (1) | 0 | 0 |
| Asian | 83 (13) | 177 (11) | 35 (16) | 84 (13) | 0 | 2 (1) | 13 (9) | 32 (8) |
| Black or African American | 34 (5) | 143 (9) | 14 (7) | 57 (9) | 12 (13) | 41 (10) | 9 (6) | 55 (13) |
| Native Hawaiian or other Pacific Islander | 3 (1) | 5 (0.3) | 0 | 2 (0.3) | 0 | 0 | 1 (1) | 1 (0.2) |
| White | 474 (72) | 1170 (69) | 161 (74) | 504 (76) | 79 (82) | 367 (88) | 124 (83) | 329 (78) |
| Multiple | 9 (1) | 21 (1) | 7 (3) | 15 (2) | 4 (4) | 4 (1) | 3 (2) | 3 (1) |
| **Ethnicity** |  |  |  |  |  |  |  |  |
| Hispanic or Latino | 300 (46) | 833 (49) | 130 (60) | 395 (60) | 54 (56) | 229 (55) | 70 (47) | 183 (44) |
| Not Hispanic or Latino | 288 (44) | 734 (44) | 81 (37) | 255 (39) | 41 (43) | 186 (44) | 80 (53) | 236 (56) |
| Not reported | 70 (11) | 120 (7) | 6 (3) | 12 (2) | 1 (1) | 4 (1) | 0 | 1 (0.2) |
| **Duration of obesity, years** | 14.2 ± 11.3 | 14.4 ± 10.6 | 18.1 ± 11.9 | 17.6 ± 11.5 | 16.5 ± 11.5 | 15.1 ± 11.1 | 15.3 ± 11.2 | 15.9 ± 12.3 |
| **Body weight, kg** | 105.0 ± 21.9 | 104.6 ± 22.1 | 99.6 ± 19.1 | 101.2 ± 21.7 | 102.2 ± 20.9 | 101.3 ± 21.2 | 106.1 ± 21.4 | 107.1 ± 22.0 |
| **Body mass index, kg/m^2^** | 38.1 ± 6.9 | 37.9 ± 6.8 | 36.1 ± 6.3 | 36.1 ± 6.7 | 35.7 ± 5.7 | 35.9 ± 6.4 | 37.9 ± 6.4 | 38.4 ± 6.4 |
| **Waist circumference, cm** | 113.7 ± 14.8 | 114.0 ± 15.3 | 114.8 ± 13.0 | 115.1 ± 14.7 | 108.7 ± 15.2 | 109.4 ± 15.0 | 115.7 ± 14.0 | 114.8 ± 14.8 |
| **Duration of type 2 diabetes, years** | N/A | N/A | 8.4 ± 7.6 | 8.6 ± 6.1 | N/A | N/A | N/A | N/A |
| **Glycated hemoglobin, %** | N/A | N/A | 7.90 ± 0.83 | 8.06 ± 0.90 | 5.30 ± 0.41 | 5.36 ± 0.39 | 5.54 ± 0.32 | 5.54 ± 0.36 |
| **Fasting serum glucose, mg/dL** | N/A | N/A | 155.6 ± 41.2 | 160.3 ± 47.3 | 89.8 ± 10.6 | 92.4 ± 10.6 | N/A | N/A |
| **Systolic blood pressure, mmHg** | 126.2 ± 12.4 | 122.3 ± 12.6 | 132.9 ± 11.5 | 129.9 ± 12.3 | 125.2 ± 11.3 | 119.8 ± 12.8 | 130.5 ± 11.6 | 124.8 ± 13.3 |
| **Diastolic blood pressure, mmHg** | 80.9 ± 7.8 | 79.1 ± 8.2 | 80.5 ± 8.2 | 79.5 ± 8.5 | 80.0 ± 9.0 | 77.9 ± 9.1 | 81.9 ± 7.6 | 80.5 ± 8.4 |
| **Pulse rate, bpm** | 71.5 ± 9.6 | 72.5 ± 9.6 | 74.6 ± 10.3 | 75.8 ± 9.9 | 69.0 ± 10.3 | 71.4 ± 10.6 | 73.0 ± 9.4 | 71.7 ± 9.3 |
| **eGFR (CKD-EPI), mL/min/1.73 m^2^** | 99.1 ± 17.8 | 97.4 ± 17.8 | 95.2 ± 17.6 | 95.1 ± 18.2 | 96.3 ± 17.7 | 96.2 ± 16.6 | 97.0 ± 16.4 | 97.7 ± 17.4 |
| **Total cholesterol, mg/dL** | 176 (91, 350) | 193 (86, 410) | 162 (92, 313) | 181 (80, 350) | 172 (113, 322) | 184 (87, 336) | 174 (106, 280) | 195 (84, 454) |
| **Non-HDL-C, mg/dL** | 125 (55, 295) | 146 (50, 384) | 115 (51, 257) | 137 (27, 331) | 121 (59, 241) | 135 (52, 286) | 125 (71, 212) | 144 (24, 366) |
| Non-HDL-C <130 mg/dL, n (%) | 373 (57) | 573 (34) | 153 (71) | 298 (45) | 60 (63) | 192 (46) | 89 (59) | 149 (36) |
| Non-HDL-C ≥130 mg/dL, n (%) | 285 (43) | 1114 (66) | 64 (30) | 364 (55) | 36 (38) | 227 (54) | 61 (41) | 271 (65) |
| **HDL-C, mg/dL** | 47 (16, 126) | 47 (21, 125) | 43 (19, 79) | 42 (15, 91) | 47 (29, 118) | 47 (23, 98) | 49 (29, 86) | 50 (25, 100) |
| **LDL-C, mg/dL** | 102 (30, 256) | 116 (16, 308) | 86 (19, 213) | 99 (4, 242) | 102 (37, 222) | 110 (25, 262) | 100 (44, 179) | 117 (3, 292) |
| **VLDL-C, mg/dL** | 52 (14, 183) | 59 (16, 179 | 64 (20, 183) | 73 (19, 177) | 45 (20, 114) | 50 (19, 163) | 58 (19, 183) | 52 (19, 169) |
| **Triglycerides, mg/dL** | 116 (31, 1950) | 131 (34, 2845) | 140 (45, 480) | 162 (42, 1896) | 97 (43, 248) | 113 (39, 480) | 124 (38, 513) | 115 (40, 614) |
| **Prediabetes** | 259 (39) | 690 (41) | N/A | N/A | N/A | N/A | N/A | N/A |
| **Hypertension** | 192 (29) | 564 (33) | N/A | N/A | 26 (27) | 155 (37) | 60 (40) | 146 (35) |
| **Dyslipidemia** | 180 (27) | 518 (31) | N/A | N/A | 20 (21) | 116 (28) | 46 (31) | 139 (33) |
| **Obstructive sleep apnea** | 49 (7) | 131 (8) | N/A | N/A | 6 (6) | 47 (11) | 20 (13) | 47 (11) |
| **Atherosclerotic cardiovascular disease** | 27 (4) | 46 (3) | N/A | N/A | 1 (1) | 9 (2) | 10 (7) | 24 (6) |

Data are mean ± standard deviation or n (%). Lipids are median (min, max). Note: Data are from all randomized participants who took at least one dose of study drug (modified intention-to-treat population) from the efficacy analysis set.

Abbreviations: CKD-EPI=chronic kidney disease epidemiology collaboration; eGFR=estimated glomerular filtration rate; HDL-C=high-density lipoprotein cholesterol; LDL-C=low-density lipoprotein cholesterol; N/A=not available or applicable; VLDL-C=very low-density lipoprotein cholesterol.
