# Supplementary material for: Achieving the triple endpoint of body weight reduction thresholds, systolic blood pressure reduction ≥5 mmHg and non-HDL cholesterol <130 mg/dL with tirzepatide in people with obesity: A post hoc analysis from the SURMOUNT trials
Source: PLoS One. 2026 Aug 13;21(8):e0345032. doi: 10.1371/journal.pone.0345032 (PMC13472364; doi:10.1371/journal.pone.0345032)
Supplement: Supplementary file 3 [file pone.0345032.s004.docx]

**S2 TABLE. Baseline demographics and clinical characteristics in participants who achieved the triple composite endpoint with body weight reduction ≥10% (Yes, No)**

| **Parameter** | **SURMOUNT-1** | | **SURMOUNT-2** | | **SURMOUNT-3** | | **SURMOUNT-4** | |
| --- | --- | --- | --- | --- | --- | --- | --- | --- |
|  | **Yes (N=603)** | **No (N=1742)** | **Yes (N=178)** | **No (N=701)** | **Yes (N=92)** | **No (N=423)** | **Yes (N=136)** | **No (N=434)** |
| **Age, years** | 44.2 ± 12.7 | 45.3 ± 12.2 | 53.6 ± 11.0 | 54.4 ± 10.2 | 43.3 ± 12.9 | 46.3 ± 11.9 | 49.0 ± 12.9 | 47.7 ± 12.6 |
| **Sex** |  |  |  |  |  |  |  |  |
| Male | 180 (30) | 579 (33) | 79 (44) | 352 (50) | 38 (41) | 151 (36) | 37 (27) | 133 (31) |
| Female | 423 (70) | 1163 (67) | 99 (56) | 349 (50) | 54 (59) | 272 (64) | 99 (73) | 301 (69) |
| **Race** |  |  |  |  |  |  |  |  |
| American Indian or Alaska Native | 45 (8) | 181 (10) | 0 | 0 | 1 (1) | 5 (1.2) | 0 | 0 |
| Asian | 76 (13) | 184 (11) | 29 (16) | 90 (13) | 0 | 2 (1) | 11 (8) | 34 (8) |
| Black or African American | 32 (5) | 145 (8) | 10 (6) | 61 (9) | 11 (12) | 42 (10) | 8 (6) | 56 (13) |
| Native Hawaiian or other Pacific Islander | 3 (1) | 5 (0.3) | 0 | 2 (0.3) | 0 | 0 | 1 (1) | 1 (0.2) |
| White | 439 (73) | 1205 (69) | 133 (75) | 532 (76) | 76 (83) | 370 (88) | 113 (83) | 340 (78) |
| Multiple | 8 (1) | 22 (1) | 6 (3) | 16 (2) | 4 (4) | 4 (1) | 3 (2) | 3 (1) |
| **Ethnicity** |  |  |  |  |  |  |  |  |
| Hispanic or Latino | 274 (45) | 859 (49) | 106 (60) | 419 (60) | 51 (55) | 232 (55) | 65 (48) | 188 (43) |
| Not Hispanic or Latino | 265 (44) | 757 (44) | 67 (38) | 269 (38) | 40 (44) | 187 (44) | 71 (52) | 245 (57) |
| Not reported | 64 (11) | 126 (7) | 5 (3) | 13 (2) | 1 (1) | 4 (1) | 0 | 1 (0.2) |
| **Duration of obesity, years** | 14.3 ± 11.4 | 14.4 ± 10.6 | 18.5 ± 11.7 | 17.5 ± 11.5 | 16.4 ± 11.3 | 15.1 ± 11.1 | 15.5 ± 11.2 | 15.8 ± 12.3 |
| **Body weight, kg** | 104.4 ± 21.5 | 104.8 ± 22.2 | 100.3 ± 19.5 | 100.9 ± 21.5 | 102.3 ± 21.0 | 101.3 ± 21.2 | 105.7 ± 21.8 | 107.2 ± 21.9 |
| **Body mass index, kg/m^2^** | 38.0 ± 6.6 | 38.0 ± 6.9 | 36.4 ± 6.3 | 36.1 ± 6.7 | 35.7 ± 5.7 | 35.9 ± 6.4 | 38.0 ± 6.5 | 38.4 ± 6.4 |
| **Waist circumference, cm** | 113.3 ± 14.4 | 114.2 ± 15.4 | 115.3 ± 13.2 | 114.9 ± 14.6 | 108.9 ± 15.2 | 109.4 ± 15.0 | 115.8 ± 14.2 | 114.8 ± 14.7 |
| **Duration of type 2 diabetes, years** | N/A | N/A | 8.1 ± 6.9 | 8.7 ± 6.4 | N/A | N/A | N/A | N/A |
| **Glycated hemoglobin, %** | N/A | N/A | 7.83 ± 0.80 | 8.07 ± 0.90 | 5.30 ± 0.41 | 5.36 ± 0.39 | 5.55 ± 0.33 | 5.53 ± 0.36 |
| **Fasting serum glucose, mg/dL** | N/A | N/A | 154.7 ± 40.1 | 160.3 ± 47.2 | 89.8 ± 10.7 | 92.3 ± 10.6 | N/A | N/A |
| **Systolic blood pressure, mmHg** | 126.1 ± 12.5 | 122.5 ± 12.6 | 132.8 ± 11.5 | 130.0 ± 12.3 | 125.4 ± 11.4 | 119.8 ± 12.7 | 130.5 ± 11.4 | 125.0 ± 13.3 |
| **Diastolic blood pressure, mmHg** | 80.8 ± 7.8 | 79.2 ± 8.2 | 80.9 ± 8.3 | 79.5 ± 8.5 | 80.5 ± 8.8 | 77.8 ± 9.1 | 81.9 ± 7.5 | 80.5 ± 8.4 |
| **Pulse rate, bpm** | 71.6 ± 9.7 | 72.5 ± 9.5 | 74.8 ± 9.9 | 75.7 ± 10.1 | 69.5 ± 10.2 | 71.2 ± 10.7 | 72.8 ± 9.2 | 71.8 ± 9.3 |
| **eGFR (CKD-EPI), mL/min/1.73 m^2^** | 99.0 ± 17.7 | 97.5 ± 17.8 | 95.4 ± 17.8 | 95.1 ± 18.1 | 96.6 ± 17.9 | 96.1 ± 16.5 | 97.2 ± 16.3 | 97.7 ± 17.5 |
| **Total cholesterol, mg/dL** | 177 (91, 350) | 193 (86, 410) | 164 (92, 313) | 179 (80, 350) | 173 (113, 322) | 184 (87, 336) | 176 (106, 280) | 194 (84, 454) |
| **Non-HDL-C, mg/dL** | 126 (55, 295) | 144 (50, 384) | 118 (51, 257) | 134 (27, 331) | 123 (59, 241) | 134 (52, 286) | 126 (71, 212) | 143 (24, 366) |
| Non-HDL-C <130 mg/dL, n (%) | 338 (56) | 608 (35) | 119 (67) | 332 (47) | 56 (61) | 196 (46) | 77 (57) | 161 (37) |
| Non-HDL-C ≥130 mg/dL, n (%) | 265 (44) | 1134 (65) | 59 (33) | 369 (53) | 36 (39) | 65 (44) | 59 (43) | 273 (63) |
| **HDL-C, mg/dL** | 47 (21, 126) | 47 (16, 125) | 44 (19, 79) | 42 (15, 91) | 47 (29, 118) | 47 (23, 98) | 49 (29, 86) | 50 (25, 100) |
| **LDL-C, mg/dL** | 102 (30, 256) | 116 (16, 308) | 88 (19, 213) | 98 (4, 242) | 102 (37, 222) | 110 (25, 262) | 100 (44, 179) | 116 (3, 292) |
| **VLDL-C, mg/dL** | 52 (14, 183) | 59 (16, 179) | 64 (20, 183) | 73 (19, 177) | 46 (20, 114) | 50 (19, 163) | 58 (19, 183) | 52 (19, 169) |
| **Triglycerides, mg/dL** | 116 (31, 1950) | 131 (34, 2845) | 141 (45, 480) | 161 (42, 1896) | 100 (43, 248) | 112 (39, 480) | 124 (38, 513) | 115 (40, 614) |
| **Prediabetes** | 236 (39) | 713 (41) | N/A | N/A | N/A | N/A | N/A | N/A |
| **Hypertension** | 178 (30) | 578 (33) | N/A | N/A | 24 (26) | 157 (37) | 53 (39.0) | 153 (35) |
| **Dyslipidemia** | 162 (27) | 536 (31) | N/A | N/A | 19 (21) | 117 (28) | 40 (29.4) | 145 (33) |
| **Obstructive sleep apnea** | 41 (7) | 139 (8) | N/A | N/A | 5 (5) | 48 (11) | 18 (13) | 49 (11) |
| **Atherosclerotic cardiovascular disease** | 23 (4) | 50 (3) | N/A | N/A | 1 (1) | 9 (2) | 9 (7) | 25 (6) |

Data are mean ± standard deviation or n (%). Lipids are median (min, max). Note: Data are from all randomized participants who took at least one dose of study drug (modified intention-to-treat population) from the efficacy analysis set.

Abbreviations: CKD-EPI=chronic kidney disease epidemiology collaboration; eGFR=estimated glomerular filtration rate; HDL-C=high-density lipoprotein cholesterol; LDL-C=low-density lipoprotein cholesterol; N/A=not available or applicable; VLDL-C=very low-density lipoprotein cholesterol.
