# Supplementary material for: Achieving the triple endpoint of body weight reduction thresholds, systolic blood pressure reduction ≥5 mmHg and non-HDL cholesterol <130 mg/dL with tirzepatide in people with obesity: A post hoc analysis from the SURMOUNT trials
Source: PLoS One. 2026 Aug 13;21(8):e0345032. doi: 10.1371/journal.pone.0345032 (PMC13472364; doi:10.1371/journal.pone.0345032)
Supplement: Supplementary file 4 [file pone.0345032.s005.docx]

**S3 TABLE. Baseline demographics and clinical characteristics in participants who achieved the triple composite endpoint achieved with body weight reduction ≥15% (Yes, No)**

| **Parameter** | **SURMOUNT-1** | | **SURMOUNT-2** | | **SURMOUNT-3** | | **SURMOUNT-4** | |
| --- | --- | --- | --- | --- | --- | --- | --- | --- |
|  | **Yes (N=516)** | **No (N=1829)** | **Yes (N=141)** | **No (N=738)** | **Yes (N=77)** | **No (N=438)** | **Yes (N=120)** | **No (N=450)** |
| **Age, years** | 43.8 ± 12.5 | 45.4 ± 12.3 | 53.5 ± 11.0 | 54.3 ± 10.3 | 43.1 ± 11.9 | 46.3 ± 12.2 | 48.9 ± 12.8 | 47.8 ± 12.7 |
| **Sex** |  |  |  |  |  |  |  |  |
| Male | 140 (27) | 619 (34) | 60 (43) | 371 (50) | 27 (35) | 162 (37) | 34 (28) | 136 (30) |
| Female | 376 (73) | 1210 (66) | 81 (57) | 367 (50) | 50 (65) | 276 (63) | 86 (72) | 314 (70) |
| **Race** |  |  |  |  |  |  |  |  |
| American Indian or Alaska Native | 40 (8) | 186 (10) | 0 | 0 | 1 (1) | 5 (1) | 0 | 0 |
| Asian | 59 (11) | 201 (11) | 19 (14) | 100 (14) | 0 | 2 (1) | 8 (7) | 37 (8) |
| Black or African American | 29 (6) | 148 (8) | 8 (6) | 63 (9) | 9 (12) | 44 (10) | 6 (5) | 58 (13) |
| Native Hawaiian or other Pacific Islander | 3 (1) | 5 (0.3) | 0 | 2 (0.3) | 0 | 0 | 1 (1) | 1 (0.2) |
| White | 378 (73) | 1266 (69) | 108 (77) | 557 (76) | 64 (83) | 382 (87) | 102 (85) | 351 (78) |
| Multiple | 7 (1) | 23 (1) | 6 (4) | 16 (2) | 3 (4) | 5 (1) | 3 (3) | 3 (1) |
| **Ethnicity** |  |  |  |  |  |  |  |  |
| Hispanic or Latino | 232 (45) | 901 (49) | 86 (61) | 439 (60) | 41 (53) | 242 (55) | 60 (50) | 193 (43) |
| Not Hispanic or Latino | 236 (46) | 786 (43) | 50 (36) | 286 (39) | 35 (46) | 192 (44) | 60 (50) | 256 (57) |
| Not reported | 48 (9) | 142 (8) | 5 (4) | 13 (2) | 1 (1) | 4 (1) | 0 | 1 (0.2) |
| **Duration of obesity, years** | 14.2 ± 11.4 | 14.4 ± 10.6 | 18.9 ± 12.0 | 17.5 ± 11.5 | 16.7 ± 10.8 | 15.1 ± 11.2 | 15.6 ± 10.9 | 15.8 ± 12.3 |
| **Body weight, kg** | 104.2 ± 21.3 | 104.8 ± 22.3 | 100.2 ± 19.0 | 100.9 ± 21.5 | 102.0 ± 22.1 | 101.4 ± 21.0 | 106.0 ± 20.2 | 107.1 ± 22.3 |
| **Body mass index, kg/m^2^** | 38.0 ± 6.4 | 38.0 ± 6.9 | 36.4 ± 6.1 | 36.1 ± 6.7 | 35.8 ± 5.9 | 35.9 ± 6.4 | 38.0 ± 6.2 | 38.4 ± 6.4 |
| **Waist circumference, cm** | 113.2 ± 14.2 | 114.1 ± 15.4 | 115.6 ± 13.0 | 114.9 ± 14.5 | 108.9 ± 16.0 | 109.4 ± 14.8 | 116.2 ± 12.9 | 114.7 ± 14.9 |
| **Duration of type 2 diabetes, years** | N/A | N/A | 7.6 ± 7.0 | 8.7 ± 6.4 | N/A | N/A | N/A | N/A |
| **Glycated hemoglobin, %** | N/A | N/A | 7.76 ± 0.77 | 8.07 ± 0.90 | 5.29 ± 0.42 | 5.36 ± 0.39 | 5.56 ± 0.32 | 5.53 ± 0.36 |
| **Fasting serum glucose, mg/dL** | N/A | N/A | 150.4 ± 40.9 | 160.9 ± 46.6 | 89.8 ± 10.6 | 92.3 ± 10.6 | N/A | N/A |
| **Systolic blood pressure, mmHg** | 125.3 ± 12.4 | 122.9 ± 12.7 | 132.7 ± 11.5 | 130.2 ± 12.2 | 124.9 ± 11.5 | 120.1 ± 12.8 | 129.9 ± 11.3 | 125.3 ± 13.4 |
| **Diastolic blood pressure, mmHg** | 80.5 ± 7.8 | 79.3 ± 8.2 | 81.0 ± 8.2 | 79.5 ± 8.5 | 80.8 ± 8.5 | 77.8 ± 9.2 | 81.8 ±7.7 | 80.6 ± 8.3 |
| **Pulse rate, bpm** | 71.6 ± 9.8 | 72.4 ± 9.5 | 74.8 ± 9.9 | 75.6 ± 10.1 | 69.9 ± 9.8 | 71.1 ± 10.7 | 72.8 ± 8.9 | 71.9 ± 9.4 |
| **eGFR (CKD-EPI), mL/min/1.73 m^2^** | 99.2 ± 17.7 | 97.5 ± 17.8 | 94.8 ± 17.3 | 95.2 ± 18.2 | 95.9 ± 17.3 | 96.3 ± 16.7 | 97.1 ± 16.3 | 97.7 ± 17.4 |
| **Total cholesterol, mg/dL** | 177 (91, 350) | 191 (86, 410) | 164 (92, 313) | 177 (80, 350) | 173 (113, 322) | 184 (87, 336) | 176 (122, 280) | 193 (84, 454) |
| **Non-HDL-C, mg/dL** | 126 (59, 295) | 143 (50, 384) | 118 (51, 257) | 132 (27, 331) | 123 (64, 241) | 134 (52, 286) | 125 (81, 212) | 143 (24, 366) |
| Non-HDL-C <130 mg/dL, n (%) | 288 (56) | 658 (36) | 91 (65) | 360 (49) | 48 (62) | 204 (47) | 68 (57) | 170 (38) |
| Non-HDL-C ≥130 mg/dL, n (%) | 228 (44) | 1171 (64) | 50 (36) | 378 (51) | 29 (38) | 234 (53) | 52 (43) | 280 (62) |
| **HDL-C, mg/dL** | 48 (21, 109) | 47 (16, 126) | 43 (19, 79) | 43 (15, 91) | 47 (29, 118) | 47 (23, 98) | 49 (32, 86) | 50 (25, 100) |
| **LDL-C, mg/dL** | 103 (30, 256) | 115 (16, 308) | 87 (19, 213) | 97 (4, 242) | 102 (51, 222) | 110 (25, 262) | 101 (44, 179) | 115 (3, 292) |
| **VLDL-C, mg/dL** | 52 (14, 183) | 59 (16, 179) | 66 (20, 183) | 73 (19, 177) | 46 (20, 105) | 50 (19, 163) | 56 (19, 183) | 52 (19, 169) |
| **Triglycerides, mg/dL** | 115 (31, 1950) | 130 (34, 2845) | 144 (45, 480) | 160 (42, 1896) | 98 (43, 229) | 111 (39, 480) | 123 (38, 513) | 116 (40, 614) |
| **Prediabetes** | 196 (38) | 753 (41) | N/A | N/A | N/A | N/A | N/A | N/A |
| **Hypertension** | 140 (27) | 616 (34) | N/A | N/A | 20 (26) | 161 (37) | 41 (34) | 165 (37) |
| **Dyslipidemia** | 133 (26) | 565 (31) | N/A | N/A | 18 (23) | 118 (27) | 35 (29) | 150 (33) |
| **Obstructive sleep apnea** | 34 (7) | 146 (8) | N/A | N/A | 3 (4) | 50 (11) | 14 (12) | 53 (12) |
| **Atherosclerotic cardiovascular disease** | 16 (3) | 57 (3) | N/A | N/A | 1 (1) | 9 (2) | 6 (5) | 28 (6) |

Data are mean ± standard deviation or n (%). Lipids are median (min, max). Note: Data are from all randomized participants who took at least one dose of study drug (modified intention-to-treat population) from the efficacy analysis set.

Abbreviations: CKD-EPI=chronic kidney disease epidemiology collaboration; eGFR=estimated glomerular filtration rate; HDL-C=high-density lipoprotein cholesterol; LDL-C=low-density lipoprotein cholesterol; N/A=not available or applicable; VLDL-C=very low-density lipoprotein cholesterol.
