# Supplementary material for: Achieving the triple endpoint of body weight reduction thresholds, systolic blood pressure reduction ≥5 mmHg and non-HDL cholesterol <130 mg/dL with tirzepatide in people with obesity: A post hoc analysis from the SURMOUNT trials
Source: PLoS One. 2026 Aug 13;21(8):e0345032. doi: 10.1371/journal.pone.0345032 (PMC13472364; doi:10.1371/journal.pone.0345032)
Supplement: Supplementary file 5 [file pone.0345032.s006.docx]

**S4 TABLE. Change from baseline in body weight, systolic blood pressure, and non-HDL-C at the primary endpoint in the SURMOUNT program**

| **Parameter** | **SURMOUNT-1 (N=2345)** | | **SURMOUNT-2 (N=879)** | | **SURMOUNT-3 (N=515)** | | **SURMOUNT-4^a^ (N=570)** | |
| --- | --- | --- | --- | --- | --- | --- | --- | --- |
|  | **Tirzepatide** | **Placebo** | **Tirzepatide** | **Placebo** | **Tirzepatide** | **Placebo** | **Tirzepatide** | **Placebo^b^** |
| **Baseline body weight, kg** | 104.7 (22.31) | 104.7 (21.29) | 100.4 (20.44) | 101.5 (22.30) | 102.7 (1.30) | 100.2 (1.33) | 106.5 (1.27) | 107.2 (1.32) |
| Change at Week 72/88 | -20.8 (0.26) | -2.8 (0.46) | -14.6 (0.36) | -3.3 (0.52) | -21.5 (0.65) | 3.0 (0.67) | -27.4 (0.50) | -10.1 (0.52) |
| Percent change at Week 72/88 | -20.1 (0.23) | -2.8 (0.42) | -14.6 (0.35) | -3.4 (0.50) | -21.2 (0.64) | 3.0 (0.66) | -25.7 (0.47) | -9.4 (0.49) |
| **Baseline systolic blood pressure, mmHg** | 123.5 (0.30) | 123.1 (0.53) | 130.4 (12.31) | 131.1 (11.83) | 121.6 (0.78) | 120.6 (0.80) | 126.8 (0.76) | 125.8 (0.79) |
| Change at Week 72/88 | -8.1 (0.27) | -1.4 (0.49) | -7.2 (0.52) | -0.9 (0.75) | -5.7 (0.73) | 4.2 (0.76) | -9.4 (0.89) | -2.3 (0.95) |
| **Baseline non-HDL-C, mg/dL** | 138.2 (0.86) | 137.2 (1.49) | 124.4 (1.65) | 129.8 (2.44) | 131.8 (2.17) | 130.3 (2.19) | 136.3 (2.17) | 134.7 (2.24) |
| Percent change at Week 72/88 | -11.3 (0.47) | -1.8 (0.96) | -6.6 (1.00) | 2.3 (1.57) | -9.8 (1.26) | 5.6 (1.55) | -10.6 (1.46) | -1.8 (1.72) |
| **Baseline non-HDL-C <130 mg/dL** | 108.0 (0.66) | 106.3 (1.15) | 97.9 (1.24) | 99.8 (1.91) | 106.7 (1.68) | 105.3 (1.67) | 107.0 (1.88) | 103.9 (1.92) |
| Percent change at Week 72/88 | -3.9 (0.85) | 6.2 (1.75) | 3.1 (1.53) | 11.6 (2.50) | -1.9 (2.05) | 14.1 (2.45) | -3.7 (3.31) | 5.3 (3.96) |
| **Baseline non-HDL-C ≥130 mg/dL** | 163.7 (0.82) | 161.8 (1.39) | 163.5 (1.61) | 164.3 (2.16) | 160.5 (2.14) | 160.8 (2.22) | 162.2 (1.93) | 162.0 (2.01) |
| Percent change at Week 72/88 | -16.0 (0.56) | -6.8 (1.11) | -16.1 (1.28) | -6.8 (1.94) | -17.0 (1.53) | -2.0 (1.93) | -16.2 (1.73) | -5.5 (2.10) |

Data are mean (standard deviation) at baseline and least squares mean (standard error) change from baseline at the primary endpoint in participants treated with tirzepatide or placebo, unless otherwise noted. Lipid data are estimate (standard error) using log transformation. The primary endpoint was Week 72 in SURMOUNT-1, SURMOUNT-2 and SURMOUNT-3 and Week 88 in SURMOUNT-4.

Note: Tirzepatide groups refer to pooled tirzepatide 5/10/15mg in SURMOUNT-1, pooled tirzepatide 10/15mg in SURMOUNT-2, and maximum tolerated doses of 10 or 15 mg in SURMOUNT-3 and SURMOUNT-4.

^a^ SURMOUNT-4 was a randomized withdrawal study with a 36-week, open-label tirzepatide lead-in period followed by a 52-week, double-blind, placebo-controlled period. Participants who completed the 36-week lead-in period experienced a mean weight reduction of 20.9%.

^b^ Placebo following an initial 36-week lead-in with tirzepatide.

Abbreviations: Non-HDL-C=non-high-density lipoprotein cholesterol.
