# Supplementary material for: Achieving the triple endpoint of body weight reduction thresholds, systolic blood pressure reduction ≥5 mmHg and non-HDL cholesterol <130 mg/dL with tirzepatide in people with obesity: A post hoc analysis from the SURMOUNT trials
Source: PLoS One. 2026 Aug 13;21(8):e0345032. doi: 10.1371/journal.pone.0345032 (PMC13472364; doi:10.1371/journal.pone.0345032)
Supplement: Supplementary file 6 [file pone.0345032.s007.docx]

**S5 TABLE. Proportion of participants achieving the triple composite endpoint across the SURMOUNT program**

| **Study** | **Body weight reduction threshold**  **Treatment** | **Observed n/N (%)** | **Imputed m/M (%)** | **LSM (SE)** | **Odds Ratio (95% CI)** | **p-value** |
| --- | --- | --- | --- | --- | --- | --- |
| **SURMOUNT-1** | **Body weight reduction ≥5%** |  |  |  |  |  |
|  | Tirzepatide 5 mg | 173/522 (33.14) | 192/594 (32.32) | 29.00 (1.97) | 6.87 (4.75, 9.93) | p<0.001 |
|  | Tirzepatide 10 mg | 179/520 (34.42) | 200/584 (34.25) | 32.37 (2.07) | 8.05 (5.56, 11.65) | p<0.001 |
|  | Tirzepatide 15 mg | 201/520 (38.65) | 223/586 (38.05) | 35.95 (2.13) | 9.43 (6.53, 13.62) | p<0.001 |
|  | Placebo | 37/463 (7.99) | 43/581 (7.40) | 5.61 (0.88) | -- | -- |
|  | **Body weight reduction ≥10%** |  |  |  |  |  |
|  | Tirzepatide 5 mg | 159/522 (30.46) | 175/594 (29.46) | 26.24 (1.89) | 8.93 (5.86, 13.61) | p<0.001 |
|  | Tirzepatide 10 mg | 166/520 (31.92) | 183/584 (31.34) | 29.42 (2.00) | 10.46 (6.86, 15.97) | p<0.001 |
|  | Tirzepatide 15 mg | 194/520 (37.31) | 216/586 (36.86) | 34.89 (2.10) | 13.45 (8.84, 20.47) | p<0.001 |
|  | Placebo | 25/463 (5.40) | 29/581 (4.99) | 3.83 (0.72) | -- | -- |
|  | **Body weight reduction ≥15%** |  |  |  |  |  |
|  | Tirzepatide 5 mg | 118/522 (22.61) | 128/594 (21.55) | 18.94 (1.64) | 10.15 (5.97, 17.26) | p<0.001 |
|  | Tirzepatide 10 mg | 156/520 (30.00) | 172/584 (29.45) | 27.98 (1.94) | 16.87 (9.96, 28.58) | p<0.001 |
|  | Tirzepatide 15 mg | 180/520 (34.62) | 200/586 (34.13) | 32.28 (2.03) | 20.70 (12.25, 34.97) | p<0.001 |
|  | Placebo | 13/463 (2.81) | 16/581 (2.75) | 2.25 (0.56) | -- | -- |
| **SURMOUNT-2** | **Body weight reduction ≥5%** |  |  |  |  |  |
|  | Tirzepatide 10 mg | 95/280 (33.93) | 100/294 (34.01) | 32.35 (2.88) | 6.52 (3.94, 10.79) | p<0.001 |
|  | Tirzepatide 15 mg | 85/263 (32.32) | 95/293 (32.42) | 29.82 (2.81) | 5.79 (3.50, 9.59) | p<0.001 |
|  | Placebo | 21/263 (7.98) | 22/292 (7.53) | 6.84 (1.44) | -- | -- |
|  | **Body weight reduction ≥10** |  |  |  |  |  |
|  | Tirzepatide 10 mg | 78/280 (27.86) | 83/294 (28.23) | 27.00 (2.69) | 13.47 (6.54, 27.76) | p<0.001 |
|  | Tirzepatide 15 mg | 78/263 (29.66) | 87/293 (29.69) | 28.36 (2.73) | 14.42 (7.00, 29.71) | p<0.001 |
|  | Placebo | 8/263 (3.04) | 8/292(2.74) | 2.67 (0.90) | -- | -- |
|  | **Body weight reduction ≥15%** |  |  |  |  |  |
|  | Tirzepatide 10 mg | 60/280 (21.43) | 65/294 (22.11) | 21.41 (2.45) | 23.57 (8.11, 68.48) | p<0.001 |
|  | Tirzepatide 15 mg | 67/263 (25.48) | 73/293 (24.91) | 24.12 (2.57) | 27.50 (9.48, 79.77) | p<0.001 |
|  | Placebo | 3/263 (1.14) | 3/292 (1.03) | 1.14 (0.60) | -- | -- |
| **SURMOUNT-3** | **Body weight reduction ≥5%** |  |  |  |  |  |
|  | Tirzepatide MTD (10 or 15 mg) | 75/223 (33.63) | 90/263 (34.22) | 32.43 (3.11) | 24.35 (10.53, 56.29) | p<0.001 |
|  | Placebo | 5/201 (2.49) | 6/252 (2.38) | 1.93 (0.78) | -- | -- |
|  | **Body weight reduction ≥10%** |  |  |  |  |  |
|  | Tirzepatide MTD (10 or 15 mg) | 74/223 (33.18) | 89/263 (33.84) | 32.07 (3.10) | 45.07 (15.15, 134.06) | p<0.001 |
|  | Placebo | 3/201 (1.49) | 3/252 (1.19) | 1.04 (0.56) | -- | -- |
|  | **Body weight reduction ≥15%** |  |  |  |  |  |
|  | Tirzepatide MTD (10 or 15 mg) | 63/223 (28.25) | 75/263 (28.52) | 27.19 (2.90) | 45.64 (12.94, 160.92) | p<0.001 |
|  | Placebo | 2/201 (1.00) | 2/252 (0.79) | 0.81 (0.51) | -- | -- |
| **SURMOUNT-4** | **Body weight reduction ≥5%** |  |  |  |  |  |
|  | Tirzepatide MTD (10 or 15 mg) | 113/298 (37.92) | 113/298 (37.92) | 35.17 (3.02) | 4.58 (2.91, 7.20) | p<0.001 |
|  | Placebo, following an initial 36-week lead-in with tirzepatide | 37/272 (13.60) | 37/272 (13.60) | 10.60 (1.90) | -- | -- |
|  | **Body weight reduction ≥10%** |  |  |  |  |  |
|  | Tirzepatide MTD (10 or 15 mg) | 108/298 (36.24) | 108/298 (36.24) | 33.22 (2.99) | 6.05 (3.69, 9.94) | p<0.001 |
|  | Placebo, following an initial 36-week lead-in with tirzepatide | 28/272 (10.29) | 28/272 (10.29) | 7.59 (1.60) | -- | -- |
|  | **Body weight reduction ≥15%** |  |  |  |  |  |
|  | Tirzepatide MTD (10 or 15 mg) | 99/298 (33.22) | 99/298 (33.22) | 30.21 (2.90) | 7.29 (4.24, 12.53) | p<0.001 |
|  | Placebo, following an initial 36-week lead-in with tirzepatide | 21/272 (7.72) | 21/272 (7.72) | 5.61 (1.36) | -- | -- |

LSM represents model-adjusted attainment rates from logistic regression. Abbreviations: CI=confidence interval; LSM=least-squares mean; M=number of participants in imputed data; m=number of participants achieving target in imputed data; MTD=maximum tolerated dose; N=number of participants with baseline value and at least one non-missing post-baseline value; n=number of participants achieving target in observed data; SE=standard error.
